# S3 Guideline: Chronic Tinnitus: German Society for Otorhinolaryngology, Head and Neck Surgery e. V. (DGHNO-KHC)

**DOI:** 10.1007/s00106-022-01207-4

**Published:** 2022-10-13

**Authors:** Birgit Mazurek, Gerhard Hesse, Heribert Sattel, Volker Kratzsch, Claas Lahmann, Christian Dobel

**Affiliations:** 1 Tinnituszentrum, Charité – Universitätsmedizin Berlin, Charitéplatz 1, 10117 Berlin, Germany; 2 Tinnitus-Klinik, KH Bad Arolsen, Große Allee 50, 34454 Bad Arolsen, Germany; 3 Universität Witten/Herdecke, Witten, Germany; 4 Klinikum rechts der Isar, Klinik und Poliklinik für Psychosomatische Medizin und Psychotherapie, Technical University of Munich, Langerstr. 3, 81675 Munich, Germany; 5 Abt. Hörbehinderung, Tinnitus und Schwindelerkrankungen, VAMED Rehaklinik Bad Grönenbach, Sebastian-Kneipp-Allee 3–5, 87730 Bad Grönenbach, Germany; 6 Klinik für Psychosomatische Medizin und Psychotherapie, Universitätsklinikum Freiburg, Hauptstr. 8, 79104 Freiburg, Germany; 7 Klinik für Hals-, Nasen- und Ohrenheilkunde, Universitätsklinikum Jena, Am Klinikum 1, 07747 Jena, Germany

## Participating professional societies/organisations

German College of Psychosomatic Medicine (DKPM)

German Society for Psychosomatic Medicine and Medical Psychotherapy (DGPM)

German Study Centre for ENT (DSZ-HNO)

German Medical Society for Behavioural Therapy (DÄVT)

German Society for Behavioural Medicine and Behaviour Modification (DGVM)

German Society for Psychiatry and Psychotherapy,

Psychosomatics and Neurology (DGPPN)

German Society for Psychology (DGPs)

German Society for Dental, Oral and Maxillofacial Medicine (DGZMK) and

German Society for Functional Diagnostics and Therapy (DGFDT)

German Society for Phoniatrics and Paedaudiology (DGPP)

German Society for Physical and Rehabilitation Medicine (DGPRM)

German Society for Paediatrics and Adolescent Medicine (DGKJ)

German Society for Audiology (DGA)

Working Group of German-Speaking Audiologists, Neuro-otologists and Otologists (ADANO)

German Professional Association of Otolaryngologists e. V.

German Tinnitus League e. V. (DTL)

European Tinnitus Network (EUTINNET)

German Association for the Hard of Hearing (DSB)

German Cochlear Implant Society (DCIG)

## Contents

What’s new?

The most important recommendations at a glance

1: Scope and purpose
1.1: Objectives and research questions
1.2: Area of care
1.3: Patients/target patient group
1.4: Addressees
2: Pathophysiological aspects and classification of chronic tinnitus
2.1: Objective tinnitus/subjective tinnitus
2.2: Time course: chronic
2.3: Possible comorbidities
2.4: Severity
3: Medical diagnostics
3.1: Anamnesis
3.2: Basic diagnostics
3.3: Further diagnostics
4: Therapy of chronic tinnitus
4.1: Presentation of therapies with recommendations
4.1.1: Tinnitus counselling
4.1.2: Interventions for hearing loss
4.1.2.1: Hearing aids
4.1.2.2: Noise generators or noisers
4.1.2.3: Cochlear implant
4.1.2.4: Hearing therapy
4.1.3: Behavioural therapy and psychodynamically oriented methods
4.1.4: Tinnitus retraining therapy
4.1.5: Music therapy approaches and sound therapy
4.1.5.1: Music therapy
4.1.5.2: Tailor-made notched music therapy
4.1.5.3: Sound therapy
4.1.5.4: Acoustic neuromodulation
4.1.6: Pharmacological treatment
4.1.6.1: Betahistine
4.1.6.2: Antidepressants
4.1.6.3: Benzodiazepines
4.1.6.4: Ginkgo biloba
4.1.6.5: Zinc
4.1.6.6: Melatonin
4.1.6.7: Oxytocin
4.1.6.8: Steroids
4.1.6.9: Drug influence on neurotransmission
4.1.6.10: Cannabis
4.1.7: Repetitive transcranial magnetic stimulation
4.1.8: Electrostimulation
4.1.8.1: Transcranial electrical stimulation
4.1.8.2: Vagus nerve stimulation
4.1.8.3: Bimodal acoustic and electrical stimulation
4.1.8.4: Invasive electrical stimulation
4.1.8.5: Intracochlear electrical stimulation—see cochlear implant
4.1.8.6: Transcutaneous electrical nerve stimulation (TENS)
4.1.8.7: Low-level laser therapy
4.1.9: Manual medical and physiotherapeutic therapy
4.1.10: Nutritional supplements
4.1.11: Acupuncture
4.1.12: Self-help
4.2: Conclusion

5: Appendix
5.1: Appendix 1: Treatment algorithm for chronic tinnitus
5.2: Appendix 2: Counselling for tinnitus
5.3: Appendix 3: Medical history questions and clinical severity classification
  References

## What is new?

The S3 guideline from 2014 was completely revised and updated. Newly introduced therapy methods were evaluated in terms of their evidence and the literature as a whole was updated (AWMF: Leitlinie Tinnitus. Leitlinien der Deutschen Gesellschaft für Hals-Nasen-Ohren-Heilkunde, Kopf- und Halschirurgie. 2021; Leitlinie 017/064: 1–108; [321]).

### The most important recommendations at a glance

Chronic tinnitus is very often associated with hearing impairment. The actual burden of tinnitus varies greatly and is largely dependent on psychosomatic comorbidities, but also on the severity of the hearing loss.

The following therapeutic interventions can be recommended:

- Counselling
- Psychotherapeutic interventions
- Hearing improvement measures

No or only very insufficient evidence is available for:

- Drug treatment of tinnitus including nutritional supplements
- Sound therapies and music therapies
- Neuromodulation such as transcranial magnetic stimulation or electrical stimulation

## 1 Scope and purpose

### 1.1 Objective and research question

Chronic tinnitus is a common symptom of the auditory system which, especially in combination with comorbidities, can lead to serious disease burden. Chronic tinnitus is not a uniform clinical picture but can take many forms. The basic medical task in chronic tinnitus are the diagnostics to identify the individually relevant factors of origin and accompanying symptoms. Therapy should be based on this differential diagnostic assessment.

The guideline presented here is intended to show the current state of diagnostics and the therapeutic concept for patients with chronic tinnitus.

### 1.2 Area of care

Physicians, dentists, psychological and medical psychotherapists, hospitals, rehabilitation facilities, health resorts.

### 1.3 Patients/target patient group

Patients with chronic tinnitus.

### 1.4 Addressees

The guideline is addressed to physicians in the fields of otorhinolaryngology, phoniatrics and paediatric audiology, psychosomatics, psychiatry, psychotherapy, paediatrics and adolescent medicine, physical medicine and rehabilitation, as well as to psychological psychotherapists, and it serves as information for neurologists, general practitioners and doctors for internal medicine.

## 2 Pathophysiological aspects and classification of chronic tinnitus

### Pathophysiological aspects of tinnitus

Tinnitus is a symptom of the auditory system. Current knowledge on the aetiopathogenesis suggests that the aetiology of tinnitus, whether symptomatic or idiopathic, may have multiple causes but is often based on a primary pathophysiological process in the inner ear. Accordingly, the frequency of tinnitus usually manifests itself in the area of the most prominent hearing loss [1, 2, 7, 8]. In the course of this pathophysiological process, among other things, highly sensitive auditory feedback mechanisms are said to be affected, which contribute to the symptom of tinnitus [3, 4]. In the case of hearing loss, for example, the cortex compensates for missing frequencies by suitable processes such as reduction of inhibitory effects—in the case of hair cell damage, for example, this is said to lead to an intensification of tinnitus and paradoxical hyperactivity of the outer hair cells [5, 6].

Central nervous processing often leads to pathologically exaggerated neuronal stimulus responses in severely affected persons with tinnitus (such as exaggerated attentional control of the tinnitus, triggering of anxiety, sleep disturbances). Particular central psychophysiological and neurophysiological processing mechanisms of the tinnitus stimulus are held responsible for these pathologically exaggerated stimulus responses.

Psychophysiologically, cognitive sensitisation [9] has been described on the cognitive level (perception level) of the brain. A primary central cause of tinnitus, however, is rare. In addition, psychosocial factors have a sensitizing influence on tinnitus perception at the cognitive level [10–12].

Neurophysiologically, changes in the neuronal firing rate, neuronal synchronicity and indications of changes in the tonotopic organisation are found in the central auditory pathway [13–17].

These changes reflect neuroplastic processes that are thought to be triggered by auditory deprivation. Similar to phantom limb pain, there is thought to be increased excitation, plasticity and connectivity along the entire central auditory pathway—as a compensatory response to reduced sensory input [18–21]. However, it has also been shown that abnormal activity can be found in somatosensory afferents [22]. Furthermore, patients with chronic tinnitus show functional changes not only in auditory structures but also in limbic, parietal and frontal areas [23–25]. The functional connectivity between auditory and non-auditory areas appears to be increased in tinnitus patients [26–31]. These tinnitus-associated structural and functional changes in neuronal networks are not static but change with increasing duration of tinnitus [26, 32, 33]. They are also significantly influenced by attentional redirection as tinnitus accentuation [34].

Activity in the auditory cortex may correlate with the subjective loudness of the tinnitus [35] but is not sufficient on its own for the conscious perception of the tinnitus. Only when abnormal activity in the auditory cortex is associated with the fronto-parietal attention network can conscious auditory perception be demonstrated [36]. The individual suffering from tinnitus is associated with the co-activation of a non-specific stress network, which includes the anterior cingulate, the anterior insula and the amygdala and which also plays a role, in addition to tinnitus, in pain syndromes and somatoform disorders [37].

Several other reviews—some of them non-recent—deal with pathophysiological aspects of tinnitus [3, 4, 9, 38–45].

The following definitions shall be used here:

### 2.1 Objective tinnitus/subjective tinnitus

The term ‘objective tinnitus’ means that there is an endogenous sound source in or near the ear whose sound emissions are heard (e.g. vascular or muscular sounds). In the strict sense, these are perceived sounds of one’s own body. In the case of ‘subjective tinnitus’, neither an external nor an endogenous sound source is present. Rather, subjective tinnitus is caused by abnormal activity in the inner ear and/or the central nervous system [40].

### 2.2 Time course: chronic

Chronic tinnitus is defined as tinnitus with a duration of at least 3 months. Depending on the justification, different time course definitions of chronic tinnitus are possible. The term ‘subacute tinnitus’ can also be found [46, 47]. For the present guideline, the definition of ‘chronic’ is justified as follows: The transitions between time courses are not static but fluid. According to the current state of knowledge, a distinction should only be made between two time courses in the choice of therapy, which will then be appropriately referred to as ‘acute’ or ‘chronic’. The time course of chronic tinnitus suggested above is therefore in agreement with the following therapy recommendations.

### 2.3 Possible comorbidities

Comorbidities (see Table 1) may be pre-existing, independent of tinnitus or tinnitus-induced. Psychological and/or psychosomatic comorbidities are frequently found in connection with tinnitus [48, 49]. In particular, anxiety disorders, depression and sleep disorders are found more frequently in patients with tinnitus. Depression and other psychological disorders are risk factors for the development of tinnitus and can intensify tinnitus [9, 50].

**Table 1:** Typical comorbidities

**1**	**Psychological/psychosomatic/psychiatric comorbidities**
*1.1*	*Affective disorders*
–	Dysthymia (ICD-10: F34.1)
–	Depressive episode (ICD 10: F32.0, F32.1, F.32.2, F32.3)
–	Recurrent depressive episodes (ICD-10: F33.0, F33.1, F33.2, F33.3)
*1.2*	*Anxiety disorder*
–	Phobic disorders (ICD 10: F40), e.g. Specific phobia (ICD-10: F40.2)
–	Anxiety disorders (ICD-10: F41), e.g. Generalized anxiety disorder (ICD-10: F41.1), Anxiety and depressive disorder, mixed (ICD-10: F41.2)
*1.3*	*Reactions to severe stress and adjustment disorders*
–	Acute stress reaction (ICD-10: F43.0)
–	Post-traumatic stress disorder (ICD-10: F43.1)
–	Adjustment disorder (ICD-10: F43.2)
*1.4*	*Somatoform disorders*
–	Somatisation disorder (ICD-10: F45.0)
–	Hypochondria disorder (ICD-10: F45.2)
–	Somatic stress disorder (Bodily distress disorder, Bodily distress syndrome [ICD-11])
*1.5*	*Psychological or behavioural factors associated with an illness classified elsewhere (ICD-10: F54)*
**2**	**Impairment of the cognitive-emotional response system**
–	Impaired concentration
–	Loss of control
–	Catastrophising
–	Resignation
–	Dysfunctional thoughts
–	Impairment of future life perspective
–	Restriction in coping with life
–	Lack of self-esteem
–	Helplessness
**3**	**Impairment of the behavioural response system**
–	Difficulty falling asleep and staying asleep
–	Social withdrawal, isolation, avoidance behaviour
–	Relationship disturbance
**4**	**Communication disorders**
–	Concomitant hearing loss
–	Recruitment
–	Disturbance of auditory perception, dysacusis
–	Hyperacusis
**5**	**Impairment of the physiological response system**
–	Myofascial imbalance in the area of the cervical spine
–	Muscle tension in the jaw and masticatory muscles, bruxism, CMD
–	Headache
–	Otalgia
–	Drowsiness
–	Vestibular disorder

The more pronounced the tinnitus distress, the more likely it is that comorbidity is present [51–53]. If psychological comorbidity is suspected, further clarification and treatment should be carried out by appropriate specialists (doctors for psychosomatic medicine, psychiatry, neurology) or psychological psychotherapists according to the existing guidelines (S3 guideline treatment of unipolar depression; S3 guideline treatment of anxiety disorders).

Comorbidities in children and adolescents are less common than in adults and differ from those of adults in terms of frequency, severity and reversibility of associated hearing loss [54–56]. Controlled studies on the frequency and severity of psychological comorbidities such as anxiety disorder and depressive episodes are not available for children. Impairments of the cognitive–emotional response system are mainly reported as concentration disorders and sleep disturbances [57].

The prevalence of tinnitus is significantly higher in patients with pain and dysfunctions of the masticatory muscles, temporomandibular joints and teeth than in patients without craniomandibular dysfunctions (CMD). There seems to be a bidirectional relationship between tinnitus and CMD so that they are assessed as respective comorbidities. Tinnitus associated with CMD and/or craniocervical symptoms is also called ‘somatosensory tinnitus’ and is classified as a subtype of subjective tinnitus because there is usually normal hearing, the average age of the patients is lower and women are over-represented [58–62].

### 2.4 Severity

The actual burden of tinnitus on a patient varies greatly; it can be determined as a degree of severity according to various criteria and is important as well as recommended for assessing the indication for therapy. The self-report instruments such as the tinnitus questionnaire ‘TF’ [63] and the short form ‘Mini-TF 12’ [64] result in a four-level classification of the degree of tinnitus distress (mild, moderate, severe, very severe). A classification of tinnitus distress suitable for everyday clinical use can be found in Appendix 3 [65].

An additional gradation of the degree of severity is the degree of compensation (often simplified in practice into the two forms of compensation–decompensation). The following applies to both degree classifications:

- Grades 1 and 2: compensated tinnitus
- Grades 3 and 4: decompensated tinnitus

This results in the following summarised description of the terms ‘compensation’ and ‘decompensation’:

Compensated tinnitus: The patient registers the ringing in the ears but can cope with it in such a way that additional symptoms do not occur. There is no or still tolerable suffering pressure. The quality of life is not significantly impaired.

Decompensated tinnitus: The ringing in the ears has a massive impact on all areas of life and leads to the development or aggravation of comorbidity (see Table 1; e.g. anxiety, sleep disorders, concentration disorders, depression). There is a high level of suffering. The quality of life is significantly impaired.

## 3 Medical diagnostics

The diagnostic process serves to determine the causes of tinnitus as well as the tinnitus distress and at the same time to clarify a simultaneously existing hearing loss. It is therefore the necessary basis for any therapeutic approach. Many factors can contribute to the development of the tinnitus symptom. In addition to otogenic causes, additional triggers and amplifying factors outside the ear must be individually determined or excluded. Diagnostics is the basis for counselling and, if necessary, therapy of the patient. Concerning what is possible and medically necessary from a cost point of view, a distinction must be made between necessary and useful diagnostics in individual cases. This should not be done in the form of a rigid scheme to be applied to every patient, but rather an individual approach determined primarily by anamnesis and basic diagnostics should be chosen.

### 3.1 Anamnesis

Anamnesis is the basis of diagnostics (!) and enables the initiation of diagnostics that are useful in individual cases. At the same time, it enables an assessment of the severity and comorbidities.

Both cause-oriented and severity-adapted diagnostics can be carried out. A structured procedure such as the evaluated ‘Structured Tinnitus Interview (STI)’ is also helpful [66].

### 3.2 Basic diagnostics

#### Clinical consensus recommendation

The basic diagnostics described should be carried out once in chronic tinnitus or when there is a significant worsening.

Classification of consensus strength: consensus (85%)

- ENT examination including tympanic membrane microscopy, nasopharyngoscopy, tube patency
- Auscultation of the ear and the carotid artery in the case of pulse-synchronous ringing in the ear or in the case of suspected objective tinnitus
- Tone audiometry, if necessary with pulsed tones, if necessary including high-pitch audiometry
- Discomfort levels, if necessary with categorical loudness scaling
- Determination of tinnitus intensity (dB HL above hearing threshold) and frequency characteristics (Hz) using narrowband noise and sinus tones
- Determination of minimum masking level (MML) using white noise and sinus tones
- Tympanometry and stapedius reflexes, optionally including recording of possible respiratory or pulse synchronous changes
- Speech audiometry without and, if necessary, with background noise: for checking a hearing aid indication, if necessary as adaptive measurement
- Transitory evoked otoacoustic emissions (TEOAE) and/or distortion products of otoacoustic emissions (DPOAE)
- Brainstem evoked response audiometry (BERA), especially in cases of unilateral tinnitus with hearing loss, cave: high stimulus levels in cases of hyperacusis.
- Orienting vestibular testing, if necessary including caloric testing and/or head impulse test
- Orienting, functional cervical spine diagnostics and examination of the dentition and masticatory apparatus in a silent environment to detect tinnitus modulations
- Blood pressure and pulse measurement

A standardised and validated questionnaire (e.g. Tinnitus Questionnaire according to Goebel & Hiller, Mini-TQ, TBF12 [63, 64, 67, 311]) is also suitable for assessing the subjective degree of severity as well as possible stress. In addition to the TQ, Mini-TQ or TBF12, the Tinnitus Handicap Inventory (THI; [68]) or, better, the evaluated version of the Tinnitus Functional Index (TFI), which is already available as a German version, should be used, especially in the context of clinical studies concerning an international comparison [69, 70].

The quantitative recording of the subjective loudness and the degree of annoyance is possible, for example, through numerical or visual analogue scales for tinnitus loudness and tinnitus distress, which can be used for monitoring the course and success of therapy [71–73].

Validated scales are preferable for international comparison [46, 63, 74].

### 3.3 Further diagnostics

#### Clinical consensus recommendation

The further diagnostics described should be carried out individually in chronic tinnitus according to the results of anamnesis and basic diagnostics.

Classification of consensus strength: consensus (93%)

Further diagnostics are to be determined individually according to the results of anamnesis and basic diagnostics. The diagnostics must be medically meaningful and economically justifiable and contribute significantly to etiological clarification, counselling and therapy.

#### 1. Extended, biographical anamnesis and/or structured tinnitus anamnesis:

in the case of a high degree of tinnitus distress (see Sect. 3.1).

The doctor (physician) or the psychologist should pay attention to symptoms of comorbid depression, anxiety disorder and/or other mental abnormalities within the framework of a psychopathological exploration since in these cases the treatment must be supplemented or adapted accordingly. They should also be sensitized to a possible masking of depressive symptoms by the symptomatology of the tinnitus to be able to take into account the complication of habituation to tinnitus by a possible tendency to depression [319].

#### 2. Dichotic test:

in the case of a disorder of the central auditory processing.

#### 3. DPOAE with contralateral stimulation:

for the detection of deficits in central inhibition.

#### 4. Diagnosis of psychological impairment, cognitive–emotional processing and coping with tinnitus (see Table 1):

in the case of a high degree of tinnitus distress.

This should be oriented towards the patient’s current complaints in connection with the tinnitus. The psychopathological diagnosis should be carried out in close cooperation with the ENT physician by a psychosomatics specialist, psychiatrist or psychotherapist experienced in tinnitus diagnostics and therapy. Anxiety and depression screening is currently done with the German version of the HADS (Hospital Anxiety and Depression Scale [75]; see guidelines Unipolar Depression, Anxiety Disorder and Depression in Children).

#### 5. Segmental examination of the muscles and joint functions of the cervical spine.

According to manual medical guidelines, range of mobility, manual medical assessment of temporomandibular joint function, muscular trigger points in the shoulder area and the masticatory musculature. X‑ray of the cervical spine, if necessary functional imaging: in the case of complaints in the cervical spine.

If the noise character (loudness, pitch, intensity, localisation) of the tinnitus can be influenced by movements and/or palpations during the initial orienting functional examination of the cervical spine and the masticatory apparatus [76], it raises the suspicion of the involvement of the musculoskeletal system. In this case, a detailed, targeted manual medical examination should be carried out to identify the structures involved and, based on the findings, to make treatment indications, exclude contraindications and prescribe adequate therapies in each case and/or involve other specialist disciplines (cf. Sect. 4.1) in the further diagnosis [77].

Independent of tinnitus modulations, patients affected by tinnitus often have concomitant complaints or pain in the cervical spine, shoulders and masticatory system [78–80]. Regarding this background, a manual medical assessment should also be considered.

#### 6. Dental functional diagnostics:

CMD screening for suspected disorders of the masticatory system.

CMD screening can reveal disorders of the masticatory system.

If there are suspicions of somatosensory tinnitus with comorbidity of craniomandibular dysfunction, there is an indication for dental functional diagnostics.

Suspicious symptoms include modulation of the tinnitus by movement or posture of the mandible, joint occurrence or intensification of the tinnitus and CMD symptoms and accompanying symptoms such as myofascial trigger points. Conservative functional therapy measures such as splint treatment and physiotherapy can have a reducing effect on the severity and intensity of the tinnitus.

Bruxism: The term ‘craniomandibular dysfunction’ includes pain and/or dysfunction of the masticatory muscles and/or the temporomandibular joints and/or dysfunction of the occlusion. The specific diagnosis is usually based on a standardized examination of the temporomandibular joints, the masticatory musculature, the mobility of the mandible as well as the horizontal/vertical jaw relation and the occlusion [81].

#### 7. Doppler ultrasonography of the brain-supplying arteries (extracranial and transcranial) and emissions close to the ear:

if there is evidence of objective pulse-synchronous ringing in the ear or clinical signs of circulatory disturbance of the brain-supplying vessels, especially provoked by head movements.

#### 8. Magnetic resonance imaging of the skull:

to clarify retrocochlear pathology, in the case of unilateral hearing loss or deafness, in the case of indications of a central auditory event, symptoms of vertigo or a neurological disease.

#### 9. High-resolution computer tomography of the petrous bones:

for detection of osseous destruction, inflammatory processes, and malformations of the petrous bone.

#### 10. Digital subtraction angiography or angiography/angio-magnetic resonance imaging (MRI)/computed tomography (CT) of the cerebrovascular system:

for pulse-synchronous tinnitus.

#### 11. Internal examination:

if diseases of the heart, circulation, metabolism or rheumatic diseases are suspected.

#### 12. Laboratory diagnostics:

in the case of suspected serological and underlying internal diseases.

- Infectious serology: e.g. Lyme disease, HIV, syphilis (see guidelines on neuroborreliosis and HIV)
- Immunopathology: immunoglobulins, rheumatoid factors, tissue-specific antibodies
- CSF diagnostics: if there is evidence of an inflammatory process of the CNS
- Metabolism: e.g. blood sugar, blood lipids, liver enzymes, thyroid hormones
- Blood count

#### 13.

In case of headache, the differential diagnosis should include trigeminal autonomic headache syndromes (e.g. migraine), space-occupying processes, pseudotumour cerebri, normal pressure hydrocephalus and abnormalities of the craniocervical junction. In the case of pulse-synchronous pulsatile tinnitus, a clarification concerning vascular anomalies (e.g. arteriovenous malformation, meningioma, aneurysm, carotid stenosis or dissection, sinus vein thrombosis) should be carried out. In these cases, a differentiated neurological or angiological diagnosis is necessary.

#### 14.

If tinnitus is associated with hearing loss and kidney disease (especially nephritis), Alport syndrome should be considered as a differential diagnosis. Certain genetic connective tissue diseases such as Ehlers-Danlos syndrome or Stickler syndrome can be associated with hearing loss and tinnitus. The diagnosis is confirmed by molecular genetics.

## 4 Therapy of chronic tinnitus

The following recommendations and evaluations refer to the therapy for chronic tinnitus that has been present for more than 3 months. For acute tinnitus, especially if it occurs in conjunction with or as a direct consequence of sudden hearing loss, reference is made to the corresponding guideline on sudden hearing loss and the systemic or intratympanic cortisone therapy recommended there [82, 83].

### 4.1 Presentation of therapies with recommendations

- 4.1.1 Counselling
- 4.1.2 Interventions for hearing loss
- 4.1.3 Behavioural therapy and psychodynamically oriented methods
- 4.1.4 Tinnitus retraining therapy
- 4.1.5 Music therapy approaches and sound therapy
- 4.1.6 Pharmacological treatment
- 4.1.7 Transcranial magnetic stimulation
- 4.1.8 Electrical stimulation
- 4.1.9 Manual medical and physiotherapeutic therapy
- 4.1.10 Nutritional supplements
- 4.1.11 Acupuncture
- 4.1.12 Self-help

Treatment is based on aetiology, severity and comorbidities. In the case of decompensated tinnitus, the result of the extended biographical, psychosomatic or psychotherapeutic anamnesis also plays an important role. In the case of chronic tinnitus, the identification of tinnitus-sensitizing causes and their therapeutic manageability as well as the long-term reduction of the patient’s tinnitus distress are in the foreground. The patient needs techniques to achieve a frequently possible desensitisation, in individual cases even a complete reduction of the tinnitus distress, to be able to deal with his or her phantom sound.

The starting point and basis of any therapeutic intervention should be counselling and education of the patient based on the diagnosis (tinnitus counselling).

#### 4.1.1 Tinnitus counselling

##### Evidence-based recommendation

Counselling should be recommended as the basis of treatment for chronic tinnitus.

Strength of evidence: 2A (moderate); level of recommendation: recommendation

Classification of consensus strength: strong consensus (100%)

Counselling should be the basis of therapy for every chronic tinnitus patient; it consists of an explanation of the findings from the diagnostics as outlined above. It also aims to avoid further comorbidities or to take into account existing ones by referring to possible interventions regarding tinnitus-specific distress.

Tinnitus counselling [84–89] is a fundamental part of the therapy of a person with chronic tinnitus (for details on the implementation, see Appendix 2).

The aim of counselling is a clarifying psychoeducational explanation and the presentation of strategies for dealing with a benign disease to reduce fears or exaggerated expectations of a cure. It is the basis for constructive habituation mechanisms and serves to avoid negative, self-reinforcing cycles in tinnitus [44, 90, 91].

The statement that there are no therapeutic options is false. The patient should be encouraged to deal with his or her ear noise in an informed way through counselling. The task of the first consulting physician is thus to advise the patient about his/her possible individual aetiopathogenesis (based on anamnesis and findings), prognosis, tinnitus-increasing factors (hyperacusis, hearing ability) or avoidance of harmful influences (e.g. noise).

If the doctor does not have the time resources for detailed counselling, they will refer the patient to a doctor or psychological psychotherapist who is qualified in tinnitus therapy. It is important for the patient, and additionally to prevent somatisation tendencies, that the treatment is not finished and that they can receive advice from the attending doctor at any time. The doctor’s role should also include counselling on alternative or new methods of treatment. This must be done based on the latest scientific knowledge. The prognostic content of counselling should be that the ringing in the ears can very often be changed by employing a suitable, non-drug therapy via a gradual reduction of the tinnitus distress and that the doctor can carry out this therapy (or refer the patient to a doctor or psychological psychotherapist in appropriately qualified practices and facilities who are qualified in suitable tinnitus therapy). As a result of successful therapy, the suffering caused by tinnitus is reduced.

Reference can be made to various manualised interventions with or without consideration of hearing loss or concomitant psychological disorders. Self-help (Sect. 4.1.12) has also been shown to be effective with guidance, although specific interventions seem to be more effective.

#### 4.1.2 Hearing loss interventions

4.1.2.1 Hearing aids

##### Evidence-based recommendation

Hearing aids should be recommended for chronic tinnitus and hearing loss.

Strength of evidence: 2b (moderate); level of recommendation: recommendation

Classification of consensus strength: strong consensus (100%), 1 abstention (conflict of interest)

Compensation of an existing hearing loss by hearing aids is a prerequisite for habituation to tinnitus and can positively influence the degree of tinnitus distress.

For the effectiveness of hearing aids in tinnitus therapy, there are only studies with moderate or weak evidence. However, this is mainly because there are practically no studies that examine the effectiveness of hearing aids alone for the treatment of chronic tinnitus. Therefore, evidence cannot be obtained, even though there are certainly indications from clinical experience that hearing aids promote tinnitus suppression and habituation. However, this requires intensive counselling and support of the patients, as a U.S. study with 133 hearing-impaired tinnitus patients proves. Hearing aid acceptance is primarily lower in the case of severely distressing tinnitus [92].

The benefit of hearing aids may be higher for low and medium tinnitus frequencies (up to 6 kHz) than for high-frequency tinnitus [93]. Other, more recent studies, however, show good effects even with isolated high-frequency hearing loss and high-frequency tinnitus [94]. In this study, 114 tinnitus patients were randomised into three groups and fitted with different types of hearing aids. Since no further counselling or education took place, a pure hearing aid effect could be demonstrated, which led to a significant improvement in the THI after 3 and 6 months. However, there were no differences between different hearing aid types or fitting strategies.

Other studies investigated differences between individual device types and found good effect sizes but no device-specific differences [95, 96]. A Swedish study investigated the effect of hearing aids on tinnitus patients, with a total of 100 patients included in the study, 50 of whom had hearing loss with tinnitus and 50 of whom had hearing loss without tinnitus. Of these 100 participants, data from 92 could finally be analysed, 46 from each group. For the patients with tinnitus and hearing loss, a significant improvement in the THI was achieved via the hearing aid application. Both groups, including the non-tinnitus patients, also improved in tests that measured cognitive functions (Reading-Span Test and Hearing-in-Noise Test; [97]).

Overall, however, there is a lack of convincing studies and meta-analyses demonstrating the effectiveness of hearing aids alone, for the systematic reasons outlined above. Accordingly, Hoare et al. [98] conclude in their Cochrane Review that a recommendation for the use of hearing aids for the indication of tinnitus cannot be made because of the poor methodology of the studies. In an update of this Cochrane Review in 2018 [99], this assessment is maintained, but overall, after evaluating studies that compared noisers (noise generators) and hearing aids, the effectiveness of hearing aids is generally confirmed.

If hearing aids are fitted, this is generally done according to the applicable national hearing aid guidelines. In individual cases of isolated high-frequency hearing loss and high-frequency tinnitus, a hearing aid fitting can be useful even without the presence of hearing loss corresponding to these guidelines [94, 100–103].

4.1.2.2 Noise generators or noisers

##### Evidence-based recommendation

Noise generators or noisers should not be recommended for chronic tinnitus.

Strength of evidence: 2a (no evidence of efficacy); level of recommendation: recommendation

Consensus strength classification: strong consensus (100%)

For tinnitus patients with hearing loss, a noiser in addition to the hearing aid is of no benefit; a mere effect of noisers in normal hearing patient has not been proven.

Noise CDs or noise generators are also discussed for tinnitus suppression, often in combination with a hearing aid [104]. A Cochrane meta-analysis evaluated six studies with a total of 553 participants and found that no improvement in tinnitus could be measured by external sounds alone or their amplification (by hearing aids). The analyzed studies stated that sound therapy was supportive. However, a clear determination of the evidence was not possible because of the multimodal therapy approaches [105]. In a review of a total of 89 studies, the high proportion of bias, caused by the commercial interests of the manufacturers and study sponsors, but also the weak methodology and often unclear definition of the outcome parameters, was criticised [106].

A Cochrane analysis from 2018 evaluated eight studies with a total of 590 participants on the effectiveness of noise therapy, mediated either by hearing aids or by sound generators. The authors criticised the fact that there was virtually no blinding and a high risk of bias in all studies. In particular, the comparison of the use of hearing aids and noisers showed no significant effects due to noiser use. Evidence of measurable superiority of sound therapy or noiser treatment over placebo or education and counselling could not be found for any device tested. Overall, the quality of the studies was weak because no second or subsequent effects on depression or anxiety were recorded. The general quality of life was also not taken into account in the studies. The authors conclude that this Cochrane analysis does not provide any evidence that noise or sound therapy for tinnitus is superior to general counselling or placebo treatment. The authors further conclude that in future studies a much better methodology with blinding and randomization and especially with the recording of other secondary outcome parameters must indeed be applied [99]. Furthermore, long-term effects of noise treatment and thus possible damage to the auditory pathway due to constant sound stimulation have not yet been investigated and thus not recorded [34, 107–109]. In studies on classic TRT, in which noisers are regularly used, data predominate with the statement that TRT produces the same effects as other counselling and habituation therapies even without the use of noisers ([110]; see Sect. 4.1.—TRT).

4.1.2.3 Cochlear implant

##### Evidence-based recommendation

Cochlear Implants (CIs) are to be recommended for profoundly deaf patients and patients with profound hearing loss, including unilaterally deafened patients with tinnitus.

Evidence strength: 2a (moderate); level of recommendation: strong recommendation

Classification of consensus strength: strong consensus (100%)

For patients with profound hearing loss and deafness, including unilateral deafness, CI fitting can provide good tinnitus suppression.

Patients with profound hearing loss or deafness may be indicated for a CI to improve their hearing. If these patients had tinnitus, an improvement is retrospectively observed more often than not [111–117]. This applies to unilateral CI implantation for homolateral [118], but also for contralateral [118] and bilateral [116, 119, 120] tinnitus. A second implant can further improve the quality of life, also concerning tinnitus [121]. Unilaterally deaf patients with very distressing tinnitus who were fitted with a CI also showed comparable results pro- and retrospectively [122–124]. The effect on the phantom sound was also independent of the tinnitus quality. Narrowband noise, tonal or even polyphonic tinnitus behaved in the same way [118].

Prospective observations of the course of tinnitus in connection with hearing loss-induced CI implantations also come to the same conclusion, although the number of study participants is still small overall [113, 119, 125]. In a prospective study of 174 CI users, 71.8% had tinnitus before surgery; in 20% it disappeared 6 months after surgery, and in 51.2% it improved [125]. Furthermore, improvements are also shown when stress processing and coping strategies are additionally assessed [116]. Exacerbations of tinnitus are possible, but remain as exceptions. In addition, there is a single non-controlled pilot study [126] with 21 CI implantations for the indication of unilateral tinnitus, from which the patients benefited significantly, according to the authors.

In individual cases with chronic tinnitus, in which the CI had only occasionally or not at all influenced the tinnitus, special electrical stimuli (biphasic with a fixed stimulation rate of 100–200 or 5000 St/s at a pleasant volume) were used. Some of the patients responded positively to at least one of the types of stimulation tested, i.e. the tinnitus was partially suppressed [127]. It was also pointed out that an electrode that is fully inserted (full length) is superior to a partial insertion [128]. However, systematic studies are lacking. At present, this procedure can still be described as experimental.

Studies on CI indications alone with the indication of tinnitus without hearing loss do not exist. Tinnitus with simultaneous occurrence of a CI-relevant—also unilateral—hearing loss can increase the indication for a CI, but cannot be the sole indication [111, 114, 115, 119, 125]. Recent studies also come to the same conclusions [129–133].

Intracochlear electrical stimulation involves delivering current to the auditory nerve via a CI electrode. The primary goal is to rehabilitate hearing in hearing-impaired patients. Some of these patients are naturally also affected by chronic tinnitus so that the effect of electrostimulation on tinnitus can be investigated in patients with a cochlear implant and tinnitus.

The 23 studies reviewed here are clinical longitudinal case studies without randomisation, and consequently, they had to be assigned to a low level of evidence (level of evidence 4). Furthermore, as a rule, on-off examinations in the period up to 1 year postoperatively were indicated, so that no statement on the long-term effect is possible. All patients were adults who had undergone CI surgery due to postlingual progressive deafness.

Various standardised questionnaires (TF, THI, TFI) were generally used to assess the outcome of tinnitus in the course before and after surgery. Some studies also recorded stress and depression indicators in addition to tinnitus. In 21 of 25 studies, a statistically significant positive effect of the CI could be determined based on the scores regarding tinnitus perception as well as the co-incidences of anxiety disorders and depression.

Among the 25 studies, there were three studies in which a comparative group was included in the observation.

In the study by Seo et al. [134], a comparison was made between tinnitus patients with a CI (*n* = 16) and a control group with an active middle ear implant (AMEI, *n* = 11) before and after surgery. As a result, surgery significantly improved THI 6 months postoperatively in 91% of patients in the group with AMEI and 56% in the group with CI. The study was only assigned a level of evidence of grade 4 due to the lack of randomisation.

Two studies could be assigned to evidence level 1b. In the first study [135], a group of 38 postlingually deaf adults were randomised. While one group received bilateral simultaneous implantation, the second group of 19 patients received sequential bilateral surgery within 2 years. Among the 38 patients, 16 had chronic tinnitus. The Tinnitus Handicap Inventory (THI) and the Tinnitus Questionnaire (TQ) were used to assess the change in subjective tinnitus perception before and after CI fitting. As a result, the preoperative tinnitus prevalence was 42%. A tendency towards a positive effect was found in both the simultaneously and sequentially operated group based on the questionnaires. There was no difference between simultaneous and sequential surgical strategies. In the group with sequentially operated patients, five persons experienced a complete suppression of tinnitus after implantation of the second side. At 2 years after unilateral implantation, two patients developed tinnitus that did not exist before the operation. This disappeared in both patients after implantation of the second side. The total follow-up time was 3 years.

In a second study [136], patients with tinnitus were also prospectively fitted with a CI either unilaterally (*n* = 19) or simultaneously bilaterally (*n* = 19). The evaluation was based on the THI, the TQ and a visual analogue scale. The prevalence of tinnitus preoperatively was 42%. After 1 year, the score in the TQ was reduced to 71.4% and in the THI to 80%. In six patients who had no tinnitus before the operation, tinnitus was induced postoperatively.

In summary, there is usually a positive effect on preoperatively existing tinnitus after a CI. Conversely, however, in rare cases, a new occurrence of tinnitus after implantation is also possible, whereby it cannot be determined with certainty whether the effect is due to the implantation or occurs spontaneously. The positive effect of a CI on tinnitus seems to be independent of age, i.e. patients over 80 years of age can also benefit from CIs for tinnitus and hearing loss.

4.1.2.4 Hearing therapy

##### Evidence-based recommendation

Hearing therapy should be recommended for chronic tinnitus.

Strength of evidence: 2a (low to weak evidence of effectiveness); level of recommendation: recommendation

Consensus strength classification: consensus (90%), two abstentions (conflict of interest)

Specific auditory therapies can promote tinnitus habituation by training and strengthening the inhibitory effects of auditory perception.

##### Minority opinion of the DGPPN.

*Hearing therapy can be considered for chronic tinnitus (recommendation level 0).*

Justification: *Due to insufficient evidence, no recommendation can be made at present. There is a need for research.*

A meta-analysis presented in 2010 shows weak evidence that auditory therapeutic approaches such as hearing therapy or audiotherapy are effective for hearing losses that can often be detected in tinnitus patients, including unilateral hearing loss as well as for primary central or psychogenic hearing losses [137].

Hearing therapy (audiotherapy) can be manualized [138]. This involves targeted exercises to improve central auditory processing skills such as directional hearing, focusing and differentiation in noise, with and without hearing aids, and specifically overhearing of tinnitus. Furthermore, auditory therapy improves the acceptance of hearing aids and can thus promote the tinnitus situation [139]. Auditory discrimination training (ADT), which requires tinnitus patients to do frequency discrimination exercises, also improves tinnitus distress [140–142]. A systematic review examined 10 studies on the effectiveness of general auditory perception training. A significant improvement in tinnitus distress was found, but all studies had low evidence due to methodological weaknesses [137, 143]. Since this review, only two relevant studies have been added: Zarenoe et al. [144] investigated the effects of additional motivational training for hearing aid fitting in tinnitus patients with hearing loss. A total of 50 patients (40–82 years old) were randomised, half of whom received the motivational training and the other half were fitted with hearing aids only. The success of the intervention was monitored with the THI. Tinnitus distress decreased significantly in both groups, but more markedly in the training group. Tugumia et al. [145] compared auditory (*n *= 6) and visual training (*n *= 6) in tinnitus patients, but could not determine any significant differences concerning tinnitus distress between the groups. However, auditory training slightly improved the THI scores, while visual training slightly worsened them.

#### 4.1.3 Behavioural therapy and psychodynamically oriented procedures

##### Evidence-based recommendation

Behavioural therapy (in various forms) is to be recommended for chronic tinnitus.

Evidence strength: 1a (high); level of recommendation: strong recommendation

Consensus strength classification: strong consensus (100%)

Extensive studies are available that prove the effectiveness of behavioural therapy interventions in comparison to waiting list control groups, but also in comparison to active control groups with regard to tinnitus distress. The effectiveness is similar for the different forms of behavioural therapy and, to a limited extent, also for internet-based behavioural therapy.

There are no indications for relevant side effects of CBT, whereas only a part of the studies systematically recorded side effects. There is no solid evidence for the long-term effects of CBT, as there are only insufficient data.

Acknowledged, psycho-physiologically based therapy methods with the goal of a tinnitus habituation form an important basis for the treatment of patients with chronic tinnitus today. They are applied on an outpatient and inpatient basis. However, it is a prerequisite that the patients are suitable for such procedures and that they can, and even must, accept the therapy and the model on which it is based.

In the case of cognitive habituation, the phantom sound still exists, but it is perceived less or is no longer perceived without active attention to the tinnitus. Habituation is the result of a specific, cognitive learning process of the brain [146]. Therefore, it is also the purpose of therapy to bring the brain from a stressful tinnitus perception as far as possible to the tinnitus habituation. This neurophysiological learning process can be described as desensitisation [147] or as habituation [41, 148]. Habituation is the goal of therapy, but an improvement in symptoms—even without reaching the final goal of complete habituation—can already represent a great gain for the person affected.

A suitable therapy method with proven effectiveness in controlled studies is structured tinnitus-specific CBT [149–154]. It aims at an improved, more indifferent handling of the tinnitus, at best again habituation.

According to a recent Cochrane Review [155] based on 28 relevant studies with a total of 2733 participants, there is (low grade) evidence that this therapy reduces the negative influence of tinnitus on quality of life. It has very few side effects and can also reduce the accompanying symptoms of depression and anxiety [155]. In this review, the effects of different forms of CBT were investigated. All participants had tinnitus for at least 3 months and their average age ranged from 43 to 70 years. The duration of CBT ranged from 3 to 22 weeks and it was mostly conducted as an outpatient intervention in clinics or online.

In some of the studies, CBT was compared only with waiting list control groups; in others, comparisons were made with active interventions (psychoeducation, auditory stimulation, TRT). The literature on behavioural therapy was systematically and meta-analytically summarised for tinnitus. The results were as follows:

**CBT versus no intervention/waiting list control**

A total of 14 studies compared CBT with no intervention/waiting list control. These studies showed that the tinnitus distress (measured with tinnitus questionnaires) was significantly reduced at the end of treatment with CBT compared to the control group (standardized mean difference [SMD]: −0.56, 95% confidence interval [CI]: −0.83–−0.30; 10 studies; 537 participants; low certainty of evidence). Converted into a score on the THI (range 0–100), this corresponds to a 10.91 point lower score in the CBT group, with the minimum clinically relevant reduction (MCID) for this scale being 7 points.

Possible adverse effects were recorded in seven of these studies: CBT probably leads to no or only minor adverse effects. Six studies reported no adverse effects and in one study one participant worsened under the CBT condition (risk ratio [RR]: 3.00; 95% CI: 0.13–69.87).

Concerning secondary endpoints, CBT may lead to a slight reduction in comorbid anxiety or depressive symptoms.

**CBT versus auditory intervention**

Three studies compared CBT with acoustic interventions. Indexed by the THI, CBT is likely to reduce tinnitus distress compared with acoustic intervention as measured by the THI (range 0–100; mean difference [MD]: −5.65; 95% CI: −9.79–−1.50; 3 studies; 444 participants; moderate certainty of evidence).

The evidence suggests that CBT can slightly reduce depression compared to auditory interventions, but there are no relevant differences in anxiety symptoms or health-related quality of life. No adverse effects were reported for either intervention.

**CBT versus tinnitus retraining therapy (TRT)**

One study (42 participants) compared CBT with TRT (including bilateral sound generators according to the TRT protocol). It was found that CBT was better at reducing tinnitus distress (measured by THI) compared to TRT (MD: −15.79; 95% CI: −27.91–−3.67; low certainty of evidence). In three participants, the values worsened during the study: once in the CBT (*n* = 22) and twice in the TRT group (*n* = 20; RR: 0.45; 95% CI: 0.04–4.64).

**CBT versus other active control**

In total, 16 studies compared CBT with another active control (e.g. relaxation, information, internet-based discussion forums). Overall, CBT can reduce tinnitus distress compared to other active treatments (SMD: −0.30; 95% CI: −0.55–−0.05; 12 studies; 966 participants; low certainty of evidence). Converted to a THI score, this corresponds to a 5.84 point lower score in the CBT group than in the other active control group. Adverse effects were systematically recorded in a few studies only. In one study, it was reported that tinnitus worsened in three participants: once in the CBT group and twice in the information-only group.

All results given refer only to the time point at the end of treatment. There is no evidence of efficacy for further follow-up time points (6 months and 12 months after the end of treatment; [155]).

Other, older studies document significant reductions in depression scores in randomised controlled trials (RCTs), presented in a meta-analysis (SMD: 0.37; 95% CI: 0.15–0.59; *I*^2^ = 0%; [156]) as well as in single RCTs [157–161].

In uncontrolled cohort studies, Brüggemann et al. [162] and Seydel et al. [163, 164] were able to demonstrate a reduction in tinnitus distress for outpatient and also inpatient therapeutic methods. Schaaf et al. [165] stress high effect sizes for inpatient tinnitus therapies.

One problem with clinical implementation is availability. The possibility of outpatient tinnitus-specific behavioural therapy exists only rarely. Here, new possibilities arise through the approval of online-based offers. In several studies, online-based behavioural therapy approaches for tinnitus were found to be as effective as classic behavioural therapy in face-to-face form, but other studies emphasise the superiority of direct face-to-face psychotherapy. Before internet-based therapy, the patient should be seen by the psychotherapist at the beginning to assess comorbidities. When considering internet-based behavioural therapies, special consideration should be given to the following: which interventions were used, the number of sessions, elements of the therapy and whether a therapist was present.

Guided self-help, as well as cognitive-oriented internet programmes (ICVT), can be alternative psychotherapeutic applications of CBT for tinnitus [160, 166]. The effect sizes are a little less smaller than with regular face-to-face CBT, but on the other hand, the so-called guided self-help, as misleadingly described by the authors, is a much more cost-effective psychotherapy than face-to-face interventions [167].

For the evaluation of internet-based behavioural therapy and tinnitus-related programmes, a systematic review and meta-analysis evaluate studies from 1990 to 2018. A total of 25 studies were included, six of which were dedicated to the treatment of tinnitus patients. Especially concerning anxiety and depression, only small effects were found in these studies. The authors conclude that internet-based CBT could be an alternative to traditional face-to-face therapy, but that the current studies cannot yet provide sufficient data certainty.

An RCT with a 2-months follow-up period investigated the effectiveness of internet-based behavioural therapy. In total, 72 patients underwent this audiologist-controlled behavioural therapy model and were compared with 73 patients who were only interviewed once a week (monitoring control group). After 2 months, the monitoring group also received internet-based behavioural therapy. Therapy success was measured with the TFI. In addition, accompanying symptoms such as insomnia, anxiety, depression and hearing loss were recorded. After the internet intervention, the reduction in tinnitus stress was significantly greater than in the control group (51% compared to 5%). This effect was seen as early as 4 weeks after the start of the therapy. In addition, the therapy also improved sleep problems, depression, hyperacusis and cognitive impairment, as well as leading to a better quality of life. Overall, the effects were followed up and remained stable 2 months after the intervention [168]. Another study by this research group randomly compared 46 patients who participated in a guided 6‑week internet-based behavioural therapy with 46 patients who received face-to-face CBT. The treatment effect (within group effect) was large in both groups (27 and 32 points reduction in TFI, respectively); the difference between the two groups (between group effect) was small, leading the authors to conclude that both interventions are similarly effective [169].

A meta-analysis examined the effect of internet-based CBT on quality of life, depression and tinnitus-related anxiety. A total of 12 RCTs with 1144 patients were analysed, each comparing psychological interventions with a waiting list. A second network meta-analysis examined 19 studies with 1543 patients in which different behavioural therapy approaches were compared with each other (“head-to-head”). Concerning tinnitus distress, face-to-face behavioural therapy, i.e. in direct contact with a psychologist, had statistically significantly the greatest potential for improvement: Tinnitus improved in 75%, depression in 83% and anxiety in 87%. Since this study compared several forms of behavioural therapy, the authors emphasise that all forms of CBT are effective therapy for tinnitus [170].

Another study compared internet-based behavioural therapy for tinnitus treatment with personalised behavioural therapy (*N* = 43). In this study, special emphasis was placed on finding out which patients were more open to internet-based offers than others. What was particularly striking here was that the patients who were open to such therapy offers also benefited more from internet therapy, but were also less burdened overall. The authors conclude that internet-based behavioural therapy can be an alternative to modern therapy approaches for open patients. However, this therapy requires independent work and a high level of self-motivation [171].

**Psychodynamically oriented methods—psychodynamic therapies**

In addition to frequently and sufficiently evaluated behavioural therapy methods, psychodynamic interventions are also used in tinnitus therapy. This happens especially within the framework of so-called multimodal therapy approaches. The condition (as for all psychotherapy methods) is that the patient and therapist must be convinced of the approach and the procedure.

Scientifically, it remains unsatisfactory that representatives of psychodynamic therapies have so far not presented any evaluated studies on the proof of evidence for tinnitus beyond casuistics.

Indirectly, the benefit of a psychodynamic approach that included cognitive–behavioural elements could be proven in an inpatient quality evaluation. From 1994 to 2007, 37 tinnitus patients were treated in an inpatient therapy setting in closed groups by the same psychotherapist in individual and group therapy over 4–8 weeks with an integrative psychodynamic behavioural therapy approach with a focus on tinnitus and hearing symptoms. This proved more effective, with an effect size of 0.93, than a comparison group treated with a serotonin reuptake inhibitor alone [172]. However, controlled RCTs and meta-analyses on the proof of efficacy regarding psychodynamic methods for the treatment of chronic tinnitus are not available.

#### 4.1.4 Tinnitus retraining therapy

##### Evidence-based recommendation

Tinnitus retraining therapy (TRT) can be considered as a long-term therapeutic intervention for chronic tinnitus. Noise generators or noisers are not required.

Strength of evidence: 1c (no evidence of efficacy for short-term use, weak evidence of efficacy for long-term use); level of recommendation: recommendation open

Classification of consensus strength: strong consensus (100%), 2 abstentions

Open recommendation only for long-term use (at least 12 months; then better than tinnitus masker) and taking into account hearing loss and hyperacusis with special attention to the counselling protocol. Due to the weak evidence of efficacy, the grading of the recommendation level only occurs with longer-term use and low effect sizes. Good evidence shows that noiser supply does not provide any additional benefit.

The core of TRT is an acoustic therapy with frequency-unmodulated noise, which was developed and introduced in the Anglo-American area based on the neurophysiological model [173–175].

TRT is a habituation technique that reduces the auditory, emotional and autonomic impact of the tinnitus noise and thus reduces the stress response to the tinnitus stimulus. It integrates three to five intervention steps including a detailed tinnitus history, auditory distraction from the tinnitus by broadband noise via a tinnitus instrument and psychological counselling.

Furthermore, TRT is a specific implementation of general tinnitus habituation therapy that uses direct counselling to reduce negative tinnitus-related reactions and the strength of the tinnitus signal [176]. As a result of tinnitus, reactions of stress, anxiety, panic attacks or loss of quality of life (fight, flight or freeze) occur. Without negative association, the fight–flight response to the tinnitus is suppressed. The main goal of TRT is to achieve habituation to the tinnitus by retraining auditory, limbic and autonomic processing in the brain [176]. This means that due to the high plasticity of the central nervous system, it is possible to reduce the response to repeated stimulation with neutral sound stimuli and by counselling [177].

In contrast to this, a working group set up in 1996 by the ADANO of the DGHNO-KHC took on the task of adapting TRT for German conditions and defining quality requirements. In the recommendation published in 2000 [178], it was suggested that sound therapy be expanded to include CBT interventions (TRT according to ADANO) and be carried out in a team by an ENT doctor, a licensed psychotherapist (doctor or psychologist) in cooperation with a hearing aid acoustician [65, 85, 86, 146, 179–181].

This form of therapy is more appropriately termed ‘tinnitus management therapy’.

Recent studies [182, 183] show that TRT with noise device therapy is not superior to treatment with placebo noise generators, although other standard treatments show success here. The specifically defined counselling sequence seems to be of particular importance in differentiating the TRT protocol from other forms of therapy for tinnitus. The effect sizes are greater for patients with decompensated tinnitus. In addition, the presence of hearing loss and hyperacusis must be given special consideration.

Studies showing that sound therapy, which goes beyond CBT in the context of TRT according to ADANO, results in an additional benefit for the patient are not available and a direct comparison of the effectiveness between TRT and CBT [184] cannot yet be made definitively due to heterogeneous outcome variables. Further studies must provide clarity here. There is no convincing proof of the effectiveness of TRT according to the evidence-based criteria required here [105]. Recent studies on ‘classic TRT’, i.e. without psychotherapeutic interventions, do not show better efficacy than other habituation therapies with counselling as an essential component. If the therapy is carried out over a longer period, the results are stable even after 18 months [182, 185, 186].

#### 4.1.5 Music therapy approaches and sound therapy

General use of tones, auditory scenes and broadband or narrowband noise in the range of the tinnitus frequency has been tested and sold in many approaches and application forms for tinnitus treatment. Noisers as apparative applications, CD or other sound carriers, and more recently smartphone-based applications have been used. Highly sophisticated methods (e.g. external biostatistician, involvement of a centre for clinical studies) and external quality management were not used.

Effectiveness could not be proven for any of the methods, or studies on this topic were not initiated at all.

4.1.5.1 Music therapy

##### Evidence-based recommendation

Music therapy approaches for chronic tinnitus can be omitted.

Strength of evidence: Ib (no proof of efficacy); level of recommendation: open recommendation

Classification of consensus strength: strong consensus (100%)

Music therapy methods are useful in terms of training hearing ability, but there are no studies that prove efficacy concerning chronic tinnitus. The gradation of the recommendation level was made due to the lack of evidence of effectiveness.

Initial clinical studies are available for three different specific forms of music therapy. For tinnitus-centred music therapy (TIM), in which the applied music is therapeutically changed within the tinnitus frequency, an application observation is available for 158 patients with acute and 18 patients with chronic tinnitus [187].

Argstatter et al. [188–191] and Grapp et al. [192] published studies on music therapy according to the Heidelberg Concept. However, a larger and statistically carefully planned study is missing to make a recommendation [193]. Furthermore, the fact that (Heidelberg) music therapy also works with behavioural therapy units and relaxation therapy is not mentioned in the studies and consequently not taken into account in the results. More recent studies are not available.

4.1.5.2 Tailor-made notched music therapy

##### Evidence-based recommendation

Tailor-made notched music therapy (TMNMT) for chronic tinnitus should not be practiced.

Strength of evidence: Ib (no proof of efficacy); level of recommendation: recommendation

Classification of consensus strength: strong consensus (100%), 2 abstentions

Music interrupted in the tinnitus frequency (notch) is offered as a smartphone app or in connection with hearing aids. It does not have a better effect on chronic tinnitus than normal, unchanged music. The gradation of the recommendation level is based on the lack of evidence of effectiveness and the possible potential for harm.

The working group of Pantev reports on person-specific filtered music applications (with recess—notch—of the tinnitus frequency, so-called tailor-made notched music therapy). The basic idea is that peripheral hearing loss leads to a reduced lateral inhibition in the range of the affected frequency, which should result in a cortical reorganisation. This maladaptive adaptation is supposed to be reversed by listening to music with an appropriate notch filter (usually half an octave). Initial evidence for this has been reported in 39 and 24 patients with chronic tinnitus, respectively, although only one study included a control population and was pseudorandomized and double-blind [194, 195]. Changes were observed in the perceived loudness of tinnitus and/or tinnitus distress. In the study by Teismann et al. [195], changes were only reported in patients with a tinnitus frequency of ≤ 8 kHz and partly only in follow-up measurements. In a subsequent study, 32 patients were divided into three groups. Two groups received either anodal (*N* = 10) or cathodal (*N* = 11) electrostimulation in combination with TMNMT. Overall, 11 patients received TMNMT and sham stimulation. All three groups reported an improvement in THQ (*p* = 0.04) when comparing baseline with post-treatment. The effect was still significant 1 month after the end of treatment. There was no modulation of the effect by electrostimulation [196].

These initial study results prompted a large-scale placebo-controlled trial of TMNMT. Stein et al. [197] published these results, in which 100 patients were randomly treated with a verum alienation (i.e. analogous to the previously determined tinnitus frequency) and 100 patients with a placebo alienation. The primary outcome measure (Tinnitus Questionnaire) did not show the predicted effect of treatment, but patients in the verum group reported a reduction in perceived loudness using a visual analogue scale (this effect was found only in the no-dropout analysis—*F* [1, 81] = 4.075; *p* = 0.047— and not in an intention-to-treat analysis). In a Chinese study, 43 patients with chronic idiopathic tinnitus were assigned to either a group receiving TMNMT or masking treatment (sound masking). The authors reported significant or highly significant changes in perceived loudness (visual analogue scale) and the Tinnitus Handicap Inventory ([198]; the publication is available in Chinese; only the abstract is available in English). Methodological aspects remain very unclear.

Furthermore, TMNMT was also studied in combination with Ginkgo biloba [199]: 26 patients were treated with the combination for 3 months. The THI score was reduced from 33.9 to 23.1 (*p* = 0.03), especially regarding the emotional component (*p* = 0.02). Due to the lack of control groups, it remains unclear in this study whether the reported effects are due to TMNMT, Ginkgo biloba or their interaction.

In the studies presented here, a reduction in tinnitus stress or a reduction in perceived loudness or a reduction of both is reported. It remains unclear where the differences come from. In the methodologically strongest study [197], only a reduction in loudness was reported, but not on the primary outcome variable (tinnitus distress). Furthermore, the patients studied suffered only from mild or moderate tinnitus.

The principle of TMNMT has now been implemented in hearing aids and tested on 20 patients with tonal tinnitus. The patients were randomly divided into a test group (*N* = 10) and a control group (*N* = 10) [200]. The test group received hearing aids with a notch filter, the control group received hearing aids without a notch filter. The outcome variable was the TQ at baseline and after 3 months. The two groups were statistically analysed independently of each other, which represents a methodological shortcoming. After 3 months, an improvement in TQ was shown for the test group (Cohen’s *d*: 0.84), but not for the control group (no statistics reported). The publication describes itself as a proof-of-concept and should be understood as such and nothing more.

There is a systematic review of internet and smartphone applications for the treatment of tinnitus [167], but only one of the studies reported above was included in the analysis [199]. This review also critically discusses the aforementioned study concerning the lack of a control group, but emphasises the improvement achieved especially in patients with tinnitus of a duration of fewer than 3 months (… ‘the improvement was good with this therapy form and is found to be more beneficial in individuals whose tinnitus is of recent onset of fewer than 3 months.’), who are then not classified as chronic according to all general criteria.

TMNMT treatment costs have even been reimbursed by some health insurances as part of a pilot project, although efficacy has not been proven in large-scale studies with external quality assurance. A survey of the 457 physicians who use this therapy also showed, with a response rate of 25.6%, that a significant improvement neither in tinnitus distress nor loudness could be achieved through notched music therapy [201].

In addition to TMNMT, another method was presented in which music was altered based on individual tinnitus characteristics. Li et al. [202] investigated the effect of listening to spectrally altered music on tinnitus. They studied 15 patients with chronic tinnitus who were asked to listen to this music for about 2 h a day for 1 year. The control group comprised 19 patients who listened to unchanged music. Changes in THI in the test group, but not in the control group, were found 3, 6 and 12 months after the start of therapy with medium effect sizes, as well as in a questionnaire for the assessment of anxiety symptoms 6 months after the start.

4.1.5.3 Sound therapy

##### Evidence-based recommendation

Sound therapy should not be practiced.

Strength of evidence: 2b (no evidence of efficacy), level of recommendation: recommendation

Classification of consensus strength: strong consensus (100%)

Various methods of stimulation by sounds, noises, auditory scenes, etc. have been proposed and tested. Due to this multitude of methods, an overall positive recommendation cannot be made. Very often, critical comparison conditions are missing and it is only shown that there are changes in the time course due to normal habituation.

Not only music but tones or complex sequences of tones and auditory scenes have been studied in various ways for the treatment of tinnitus. Durai and Searchfield [203] compared the influence of listening to natural sounds with static broadband noise in a cross-over design. Patients were randomly assigned to two groups in which the order of the listening condition was counterbalanced. The two phases lasted 8 weeks each, whereby the individual duration varied greatly. Overall, various effects were measured over time for both conditions, but as there was no untreated control group, these effects are difficult to interpret. The TFI as the primary outcome variable showed a greater reduction in the broadband noise condition, but the tinnitus loudness matching worsened. Overall, nature noise was expected to lead to improvement, but the opposite was true. In another study, broadband noise was compared as a control condition against a variant of sound therapy called ‘harmonic sound therapy’ [204]. In this therapy, sounds with narrowband noise around the first and fourth subharmonic are presented around the tinnitus frequency. A total of 18 patients were randomly assigned in a cross-over design with two arms and received the respective therapy conditions for 2 h a day for 3 months. Again, when the study arms were compared independently, several effects were seen in various variables over the time of treatment and when the arms were compared independently. When the study arms were compared directly, there was only a reduction in perceived loudness and THI. The analysis strategy has statistical flaws as not all comparison cells are included in the analysis.

Broadband noise compared to sine tones was investigated by Li et al. [205]. Overall, 14 patients each were randomly assigned to one of the two conditions and were to use the stimulation for 3 months, three times a day for 30 min. Both THI and loudness showed a reduction during therapy up to 12 months afterwards.

Simonetti et al. [206] used fractal tones, i.e. harmonic and melodic tones and tone sequences that are, however, not predictable. These tones were to be listened to for 8 h a day for 6 months. There was no control group and only six patients were reported. The small sample group showed a change in THI. Due to the small sample size and the lack of a control group, the data are difficult to interpret.

As another auditory stimulation procedure, Munro and colleagues [207] used ocean noise presented binaurally with ‘binaural beats’. The perception of binaural beats occurs when two stimuli with small frequency differences are presented simultaneously in dichotic listening. This creates the perception of a modulation. In 20 patients, the effects of this 10-min stimulation were compared with those in 20 patients with stimulation with unmodified ocean noise. No differences were found between the stimulation conditions.

Noise stimulation was also studied during sleep in tinnitus patients [208]. In total, 58 patients were randomly divided into three groups receiving either an individualized tinnitus sound or a non-individualized sound, both conditions via in-ear headphones, or a sound via a bedside device. The application was to be every night for 3 months. The application of the two conditions with in-ear headphones reduced the scores for tinnitus distress compared to the third group. The individualized noise resulted in a reduction in tinnitus loudness compared to the other two noise conditions.

Tyler et al. [209] investigated the effect of masking tinnitus compared to no masking in a cross-over design in 18 patients. The data were not statistically analysed and the description of the effects remains descriptive.

Masking of tinnitus in patients with and without hearing loss was studied by Rocha and Mondelli [210]. In each case, 15 patients were assigned to a non-randomised group and received tinnitus masking (in patients without hearing loss) or masking with a hearing aid (in patients with hearing loss) for 6 months. The authors report an improvement in THI and VAS in both groups, which cannot be assessed conclusively as crucial statistical parameters are not reported. Comparison of the groups is problematic because of confounding variables.

Sereda, Davies and Hall [211] report on a descriptive level in a feasibility study of eight patients that hearing aids with tinnitus-specific masking are applicable and user-friendly.

4.1.5.4 Acoustic neuromodulation

##### Evidence-based recommendation

Acoustic neuromodulation is to be omitted.

Evidence strength: 1c (no proof of efficacy); level of recommendation: strong recommendation

Classification of consensus strength: strong consensus (100%), 1 abstention (conflict of interest)

There is insufficient evidence for the efficacy of acoustic neuromodulation according to the CR method (coordinated reset). The recommendation was upgraded to a strong negative recommendation due to the potential for harm and the economic burden on patients.

Acoustic reset neuromodulation is a procedure that aims to achieve tinnitus reduction by presenting certain tones in the range of the individual tinnitus frequency. A first clinical pilot study on 63 patients (unilaterally blinded, randomised, placebo-controlled) showed statistically significant improvements. However, the placebo group of 5 patients was very small and their tinnitus was in a different frequency range [212]. Results of phase III studies are not available or were not allowed to be published at the request of the study sponsors [213]. An uncontrolled cohort study suggested efficacy, but also described isolated tinnitus exacerbations [214]. A systematic review concluded that the available evidence is insufficient to recommend the procedure in routine clinical practice [215]. Placebo-controlled studies are lacking [216].

#### 4.1.6 Pharmacological treatment

##### Evidence-based recommendation

The distribution of drugs for the therapy of chronic tinnitus is to be avoided.

Evidence strength: 1a–2b depending on the drug group (no proof of efficacy); level of recommendation: strong recommendation

Classification of consensus strength: strong consensus (100%)

There are insufficient data on the efficacy of drug treatments specifically for tinnitus, but evidence for potentially significant side effects. Recommendation is based on systematic reviews and randomised trials. Evidence for non-recommendation: betahistine, ginkgo, antidepressants (1a), benzodiazepines, zinc, melatonin, cannabis (2), oxytocin, steroids and gabapentin (2b). Because of the possible risk for patients, the strength of recommendation is upgraded even if the evidence for individual groups of preparations is only moderate.

The pharmacological treatment of frequent comorbidities of tinnitus, such as anxiety disorders and depression, should be distinguished from this. These comorbidities should be treated according to the available guidelines, whereby drug treatments can also be used.

Treatment of acute tinnitus follows the treatment recommendations for acute sudden hearing loss. For the treatment of acute hearing loss, corresponding guidelines (AWMF 017/2914 currently being updated [82]; guideline USA [83]) recommend systemic or intratympanic steroid treatment with, however, moderate evidence. Therefore, acute tinnitus with hearing loss or following hearing loss should be treated appropriately. If the tinnitus occurs acutely without measurable hearing loss, the standard cortisone therapy recommended for hearing loss is not recommended.

Drug therapy for chronic tinnitus:

A review from the United States essentially refers to two types of pharmacotherapies for tinnitus. The first is being researched in the hope of directly eliminating tinnitus. The second group of drugs has been developed to treat or at least alleviate possible comorbidities and thereby also improve the quality of life of tinnitus patients. While there are practically no promising approaches for the first category to eliminate tinnitus from perception, there are numerous medications that are suitable for the treatment of comorbidities; usually modern antidepressants [217]. These are used because individual psychosomatic factors can play a decisive role concerning the actual distress of the tinnitus [4].

Therapeutic approaches such as intratympanic steroid treatment do not affect chronic tinnitus [218, 219] if it does not occur in conjunction with acute hearing loss. A temporary increase in tinnitus intensity and tinnitus distress is not considered new-onset tinnitus but should be considered and treated as a fluctuation in the subjective perception of chronic tinnitus [220].

Many classes of drugs have been used or tried for chronic tinnitus, including various antiarrhythmics, anticonvulsants, anxiolytics, glutamate receptor antagonists, antidepressants, muscle relaxants and others [221], with little evidence of greater benefit than harm [222]. Experimental studies, some of them phase II or even phase III, with NMDA receptor antagonists or AMPA receptor antagonists had also shown promising approaches, but ultimately a breakthrough was not apparent, which is why these types of drugs did not reach market maturity [223].

Meta-analyses and RCTs with usable results and numerous non-usable and comparable publications are available. For some preparations, there are indications of possible efficacy in individual randomised clinical trials, but there are no replicated positive results from randomised clinical trials with sufficient evidence or positive results from meta-analyses for a single preparation. Accordingly, neither the European Medicines Agency (EMA) nor the Food and Drug Administration (FDA) has approved any preparation for the treatment of tinnitus [224]. The tinnitus guideline from Great Britain, which was updated in 2020, also made a recommendation against drug treatments, especially the drug betahistine [225].

For betahistine, Ginkgo biloba and antidepressants, Cochrane meta-analyses show no evidence for the efficacy of the respective preparations in chronic tinnitus.

4.1.6.1 Betahistine

A Cochrane Review of the use of betahistine in the treatment of tinnitus states that in England, for example, more than 100,000 prescriptions for betahistine are written out for tinnitus each month, especially by general practitioners but also by specialists. In the Cochrane Review, five studies with a total of 305 participants were evaluated. Differences in tinnitus distress and the corresponding questionnaires after treatment with betahistine or placebo were not found, nor were accompanying symptoms such as depression influenced differently by betahistine than by placebo. Thus, there is no evidence for betahistine in the treatment of chronic tinnitus [226].

4.1.6.2 Antidepressants

The Cochrane Review of antidepressants for tinnitus [227] identified six RCTs (610 patients) on this topic. Only one study was considered to be of high quality. This study compared the effect of paroxetine (SSRI—serotonin reuptake inhibitor) with placebo and showed no significant difference in effect between the groups. No effect was shown for trazodone (serotonin antagonist and reuptake inhibitor) and only a small effect was shown for tricyclic antidepressants, but this may be due to methodological problems in these studies. Side effects were frequently reported, including sedation, sexual dysfunction and dry mouth. Nevertheless, antidepressants are often used successfully in the treatment of depression and anxiety; however, not to improve tinnitus, but to treat the accompanying depression and/or anxiety symptomatology or a distressing sleep disorder.

4.1.6.3 Benzodiazepines

The systematic review by Jufas and Wood [228] on the use of benzodiazepines for tinnitus included six clinical trials that investigated the use of diazepam, oxazepam and clonazepam. There were mixed results across the studies and methodological issues, and thus this limits the assessment of effect. In summary, it was concluded that the use of benzodiazepines for subjective tinnitus does not have a robust evidence base and that these drugs should only be used as bridging drugs with strict indications, e.g. in the context of the initiation of antidepressant therapy, because of their considerable side effects (especially the short-term development of dependence).

4.1.6.4 Ginkgo biloba

Ginkgo biloba is the most commonly used herbal supplement for tinnitus. Older systematic reviews included four RCTs on Ginkgo biloba for tinnitus as a primary complaint [229]. A second systematic review included five RCTs, with most studies having low methodological rigour and conflicts of interest [230]. The results were positive for Ginkgo, but the authors stated that a clear conclusion on efficacy was not possible. A meta-analysis pooled data from six RCTs and concluded that Ginkgo has no benefit over placebo [231]. Ginkgo biloba can interact with other blood thinners to cause severe bleeding, which may increase the risk of bleeding in patients with underlying coagulation disorders [232].

A review from Norway as an update of a Cochrane Review [229] evaluated new randomised and placebo-controlled studies on the efficacy of Ginkgo biloba with a total of over 6000 patients: Evidence was found neither for efficacy in cognitive deficits, in dementia, in apoplexy, in claudicatio nor in tinnitus. Rather, there are—albeit mild—side effects such as dizziness, stomach complaints or allergic reactions, sometimes also an increased bleeding tendency [233]. As early as 2001, the efficacy of Ginkgo extract for tinnitus was investigated in the *British Medical Journal* (BMJ) in a double-blind and placebo-controlled study with a very large patient population (1121 participants). Ginkgo led to the same (small) improvement in tinnitus penetrance and intensity as a placebo [234].

Also in connection with other indications, Ginkgo extracts only showed an effect under very special circumstances. In a systematic review by Spiegel et al. [235], positive effects of EGb 761 (Ginkgo extract) compared to placebo were demonstrated in dementia in combination with tinnitus. The basic assumption for this analysis is that Ginkgo extract has a positive effect on tinnitus and vertigo symptoms, which has already been proven elsewhere. Five studies were systematically included that fulfilled the inclusion criteria: a diagnosis of dementia according to generally accepted criteria, long-term treatment of at least 20 weeks as well as simultaneous measurement of parameters of other indication areas such as the simultaneous occurrence of tinnitus and dizziness and finally an evaluation before and after the treatment. For this, five studies were identified that found EGb 761 to be significantly superior to placebo for both tinnitus and dizziness. The authors conclude that EGb 761 is also effective for neurosensory symptoms associated with dementia. Why numerous other randomised and placebo-controlled studies investigating (and failing to confirm) Ginkgo effects on tinnitus were not included in the analysis is not answered in the paper.

A meta-analysis of three reviews, on the other hand, found no influence on the severity of tinnitus and no improvement in tinnitus intensity and quality of life after administration of Ginkgo biloba [236].

The methodological comparability of the studies available so far is not given with different administration of the drug compared to placebo and different outcome variables.

4.1.6.5 Zinc

A Cochrane analysis evaluated RCTs assessing zinc against placebo in the treatment of tinnitus in adults. Three studies with a total number of 209 participants were reviewed. Improvement in tinnitus distress was assessed with questionnaires (Tinnitus Handicap Questionnaire). On the whole, there was no significant difference in any of the studies compared to placebo, neither in terms of primary treatment success nor in terms of secondary success rates. However, no side effects were described or found. None of the studies could find a significant improvement in quality of life or even an improvement in depression and anxiety in patients treated with zinc [237].

4.1.6.6 Melatonin

Melatonin is a hormone secreted by the pineal gland and is involved in the regulation of the sleep–wake cycle.

Three RCTs with a total of 193 participants have studied melatonin for the treatment of tinnitus and each has shown benefits with the greatest improvement in patients with severe tinnitus and insomnia [238]. However, given the small total number of patients studied and the methodological limitations, including the lack of a placebo group in the largest study, these results should be interpreted with caution. Although another study showed a potential benefit for patients with concurrent sleep disturbance due to tinnitus, this study lacked randomisation, blinding or placebo control [239]. Only one study reported potential side effects of melatonin, including bad dreams and fatigue [240]. A review evaluated studies investigating the treatment of tinnitus patients with melatonin. In five studies, no evidence was found for therapeutic success concerning tinnitus, but at least sleep disturbances improved [241].

4.1.6.7 Oxytocin

In a pilot study, the treatment of chronic ringing in the ears with the hormone oxytocin was investigated. Oxytocin is a hormone that, among other things, helps to regulate signal transmission in the brain and is stimulated by high doses of oestrogen. It mainly regulates contractions during birth and the production of breast milk and sperm. Social factors are also thought to be controlled by regulation in the amygdala, such as empathy and trust, but also attention to acoustic stimuli.

The study tested whether attention to chronic ear noises can also be influenced by the hormone: 15 patients with chronic tinnitus were given oxytocin as a nasal spray twice a day for 10 weeks. Five of these patients reported an improvement in their ringing in the ears—it became quieter. A second double-blind, placebo-controlled study was then conducted with 17 tinnitus patients who received only a single dose (oxytocin or placebo). Here, the patients who received the hormone reported a minimal improvement in their tinnitus 30 min to 24 h after hormone administration. Overall, there was only a very small difference compared to the placebo. In the long-term treatment (15 patients over 10 weeks), the reduction in tinnitus distress and loudness was measurable, but also not very strong and not placebo-controlled [242].

4.1.6.8 Steroids

Controlled studies on the treatment of chronic tinnitus with systemic steroid administration are not available. In a corresponding search, only 2 studies were found that used corticosteroids intratympanically, both without significant effect. One study randomised 70 adult tinnitus patients to intratympanic treatment with either methylprednisolone or saline. However, the severity of tinnitus did not change significantly in any group [218]. The other study also randomised intratympanic treatment (*n* = 36) with dexamethasone or saline, also without significant differences [243]. For a more recent study, 107 patients aged 20–77 years with idiopathic chronic tinnitus were randomised. One half received six intratympanic injections of dexamethasone, a total of twice a week for 3 weeks. The control group received saline injections. Treatment outcome was measured with the Tinnitus Handicap Inventory (THI), before treatment, after 1 week, after 1 month and after 6 months. In this study, the verum-treated group showed an improvement in THI after 6 months compared to the control group, although only just marginally significant, while hearing thresholds did not improve with this treatment [219]. Other studies with a similar study design had not found any effects in the case of long-term tinnitus, but at best in the case of acute tinnitus.

4.1.6.9 Medicinal influence on neurotransmission

##### Gabapentin.

In 72 tinnitus patients, the administration of gabapentin was investigated in comparison with placebo; a third group also received a local injection of lidocaine into the auditory canal once a week. The best effect in terms of tinnitus distress measured with the THI was found for the combination of the local anaesthetic with gabapentin; only a very small effect was found for gabapentin alone. However, the authors found no explanatory pattern as to why lidocaine may work [244].

##### Glutamate antagonists.

Four glutamate antagonists have been used in clinical trials with tinnitus patients: Acamprosite/Acamprosate, Memantine, Neramexane and Caroverine. All antagonists were used as off-label medications or before approval. There was no evaluated and reproducible therapeutic success.

4.1.6.10 Cannabis

In recent years, the use of cannabinoids has been discussed for practically all chronic diseases, and the first studies exist regarding the evidence of cannabis treatment in ENT medicine. A review examined 79 publications but discussed all possible ENT indications such as blepharospasm, alleviation of radiation side effects and psychological treatment of a cancer diagnosis. According to these authors, real evidence for a meaningful cannabis treatment is not yet given; this would have to be provided by further research [245].

One paper directly discusses the effect of cannabinoids on tinnitus [246]. It essentially refers to animal experiments that show that effects on the dorsal cochlear nucleus are to be expected in the brain through cannabis medication and thus hyperactivity and consequently an intensification of the tinnitus could be achieved. A positive or alleviating effect on ringing in the ears and tinnitus is therefore not to be expected from cannabis, at least according to the animal studies currently available.

In a recent review, connections between cannabis effects and an influence on the immune response of the auditory system are presented and a potentially positive effect for tinnitus therapy is postulated, without, however, being able to draw on studies for this [247].

*Based on the aforementioned data, no pharmaceutical can be recommended for the treatment of chronic tinnitus.* *However, if psychiatric comorbidities (e.g. anxiety disorders, depression) exist in connection with tinnitus, these should be treated. Regarding the type of treatment, reference is made to the corresponding guidelines (S3 guideline for the treatment of unipolar depression; S3 guideline for anxiety disorders).*

#### 4.1.7 Repetitive transcranial magnetic stimulation

##### Evidence-based recommendation

Transcranial magnetic stimulation methods of the auditory cortex should not be practiced in chronic tinnitus.

Strength of evidence: Ib (conflicting evidence of efficacy); level of recommendation: recommendation

Classification of consensus strength: consensus (92%)

Methods for transcranial magnetic stimulation of the auditory cortex have been extensively researched in studies with mostly very small numbers of cases: Current meta-analyses are contradictory regarding an efficacy that goes beyond placebo. A downgrading of the level of evidence and the level of recommendation occurs due to heterogeneity of the primary studies (e.g. different study protocols, individual studies of unclear efficacy) in the current meta-analyses and due to the benefit–harm assessment.

##### Minority vote of the DGPPN.

*Transcranial magnetic stimulation can be considered for the treatment of chronic tinnitus (recommendation grade 0).*

Justification: *Due to the low level of evidence, no recommendation can be made at present. There is a need for further research.*

Repetitive transcranial magnetic stimulation (rTMS) is a procedure that enables non-invasive influencing of neuronal excitability of superficially located brain areas. For about 20 years, rTMS has been investigated for the treatment of tinnitus. There are numerous randomised controlled studies with a total of over 1000 participants. In some cases, different stimulation protocols were investigated [248].

Only some of these studies showed significant efficacy of rTMS. Systemic reviews and meta-analyses all concluded that the treatment has few side effects and is safe. The study data on efficacy are heterogeneous.

A methodically good and comprehensive randomised and placebo-controlled study evaluates the therapeutic effect of 1‑Hz low-frequency repetitive transcranial magnetic stimulation for tinnitus treatment. The authors, who have many years of study experience with this type of treatment, compared the efficacy of a 2-weeks treatment with 10 sessions and 2000 stimuli each on the left temporo-parietal cortex against a comparative treatment with a sham coil. In total, 153 patients were enrolled in the study; 75 received the real treatment, and 78 the sham treatment. After the treatment, the tinnitus distress remained identical in all patients, and the accompanying symptoms such as quality of life and depression, which were also examined, showed no significant difference between the two treatment groups. Although the therapy was well tolerated overall, it did not show a therapeutic effect; the follow-up period was 6 months [249].

Systematic reviews including a Cochrane analysis concluded that short-term treatment effects are detectable, but longer-lasting effects are not proven [249, 250]. The number of studies is limited, and long-term effects (including side effects) have not been sufficiently investigated.

In a recent meta-analysis on different forms of neurostimulation, the effects of 28 randomised controlled studies on rTMS were systematically analysed [251]. It showed that active rTMS significantly reduced tinnitus distress at the end of treatment compared to sham treatment (effect size: −0.45; CI = −0.66; −0.24; *p* < 0.0001; [252]). Longer-term effects of treatment were evaluated in some studies for the period between 1 week and 6 months after treatment. These showed sustained improvements (effect size: −0.42; CI = −0.68; −0.15; *p* = 0.0024). The effects were most pronounced with stimulation of the left auditory cortex, and women responded better to treatment than men did.

A Chinese meta-analysis evaluated 22 studies with a total of 1228 patients in which rTMS was compared with sham treatment. The mean difference in THI reduction between active rTMS and sham was −7.92 (CI = −14.18; −1.66) 1 week after treatment, −8.52 (CI = −12.49; −4.55) 1 month after treatment and −6.53 (CI = −11.4; −1.86) 6 months after treatment. However, not all studies (only two) were included in the 6‑month follow-up, especially those that found no improvements [253].

Since the minimum clinically relevant reduction (MCID) for the THI scale is 7 points [254], the effects observed in the follow-up period of 6 months compared to sham treatment are consistently classified as clinically relevant.

However, the studies examined in this meta-analysis were also very heterogeneous; the results were not significant in 13 studies.

In a recent meta-analysis that also evaluated the same studies, Dong et al. [255] concluded that rTMS did not provide a significant improvement compared to sham treatment. Ten RCTs with 567 participants were analysed. Neither short-term (*p* = 0.72), nor medium-term (*p* = 0.41), nor long-term (*p* = 0.14) showed significant improvements for tinnitus distress in either the THI or the TQ.

No serious side effects were reported in any of the studies. Headaches were listed as the most frequent side effects of active rTMS. The frequency of side effects did not differ significantly between active and sham rTMS, although the difference was not significant [253].

However, it must be taken into account that these data are based only on RCTs of rTMS for tinnitus with about 1000 patients, and not all of these trials systematically reported side effects.

In summary, the efficacy of repetitive transcranial magnetic stimulation is questionable.

#### 4.1.8 Electrostimulation

4.1.8.1 Transcranial electrical stimulation

##### Evidence-based recommendation

Transcranial electrical stimulation methods should not be practiced for chronic tinnitus.

Strength of evidence: 2b (no evidence of efficacy); level of recommendation: recommendation.

Classification of consensus strength: strong consensus (100%)

There is evidence for the safety of transcranial electrical stimulation, but there is no evidence for its effectiveness in chronic tinnitus due to a lack of controlled studies and meta-analyses. Most studies display no difference between real and placebo (sham) stimulation.

In transcranial direct current stimulation (tDCS), a low current (0.5–2 mA) is applied to the cortex via the scalp. Depending on the polarity, this results in an increase or decrease in cortical excitability in the stimulated areas. Fregni et al. [256] were the first to propose this for tinnitus treatment. A review [257] included 17 studies, but only two were randomised and controlled. The meta-analysis concluded that there was insufficient evidence that tDCS was effective against tinnitus. Further RCTs of tDCS with different forms of stimulation were requested. Since then, further studies have been published, which certify that the method is safe, but that the effect on tinnitus is small to non-existent.

A Swiss study investigated the effect of transcranial direct electrical stimulation (tDCS) in a double-blind and placebo-controlled manner on 42 patients [258]. The cathode was applied via the auditory cortex, the anode was applied prefrontal. Although there were no side effects, there were no effects on tinnitus.

In another study, 40 patients were treated for 6 months either with anodal tDCS or with a so-called sham stimulation, i.e. placebo. Afterwards, all were fitted with hearing aids for 6 months. After 3 months of hearing aid use, all improved in tinnitus distress, regardless of whether they had previously received real or sham stimulation [259].

A Japanese research group investigated the influence of tDCS on connectivity [260]. Since the connectivity between the left and right auditory cortex seems to be less pronounced in tinnitus patients than in normal-hearing patients, nine tinnitus patients were compared with nine control participants. After tDCS treatment, the connectivity between the auditory cortex and somatosensory and motor brain areas decreased in the tinnitus patients, while this connection remained strong in the control group.

A total of 22 patients with tinnitus existing for more than 6 months were divided into two groups in a study; 11 were stimulated anodally with 2 mA for 20 min over left temporo-parietal areas in five sessions, and 11 patients had sham stimulation. Tinnitus loudness in VAS, THI and stress level were assessed. No significant differences were found between the anodal and sham stimulation groups. The tinnitus distress of some patients worsened or the tinnitus changed; long-term effects were not found [261].

One study compared tDCS with sham stimulation at the shoulder in 24 patients. No significant differences were found in the TFI between the two groups, not even concerning tinnitus intensity [262].

In another study, 35 patients with chronic tinnitus were treated with tDCS for 10 days for 20 min each, either left temporal or bifrontal stimulation or only with sham. All three groups improved in THI scores, but differences between groups were not found [263].

In a double-blind placebo-controlled study, 25 patients were treated with tDCS in 10 sessions, and 15 patients received sham treatment. After 1 month, various data were evaluated, in particular THI and VAS scores. Some side effects were also accurately recorded, especially itching, but without any serious side effects. However, the paper only describes the data collected concerning future study protocols; specific results were not reported [264].

A meta-analysis summarised the results of 32 RCTs and found a positive effect, especially for cathodal transcranial direct current treatment of the cortex, concerning the severity of tinnitus and the improvement of quality of life. However, measuring instruments were not described and outcome parameters were not evaluated reliably; long-term observations were also not available. Four studies investigated tDCS, and two compared tDCS with tRNS (transcranial random noise stimulation). Only in two (older) studies was the outcome with tDCS better than sham stimulation [265].

Out of a total of 85 studies on tDCS, 34 were evaluated in another review [266]: Here too, however, mainly the very different forms of application and study designs were described; an evaluation of the concrete therapy results of the individual studies was not undertaken. It is emphasised in this review that tDCS is often mixed with other methods such as various forms of acoustic stimulation in particular, which makes an overall evaluation difficult.

4.1.8.2 Vagus nerve stimulation

##### Evidence-based recommendation

Transcutaneous or invasive vagus nerve stimulation alone or in combination with acoustic stimulation should not be practiced for chronic tinnitus.

Strength of evidence: 2b (no proof of efficacy); level of recommendation: recommendation

Classification of consensus strength: strong consensus (100%)

Transcutaneous vagus nerve stimulation, as well as invasive cervical implantation, is safe to use, but evidence for efficacy in chronic tinnitus is not available.

Vagus nerve stimulation is thought to stimulate cholinergic basal nuclei, which are assumed to be responsible for extensive changes in cortical organisation. This is hypothesized to improve learning effects, as has been found in animal studies. Tinnitus patients should be acoustically stimulated to ‘unlearn’ their tinnitus. Stimulation of the vagus nerve should then support this learning [267]. Vagus nerve stimulation can be done invasively by an implanted vagus nerve stimulator or non-invasively as transcutaneous vagus nerve stimulation by electrostimulation of the external auditory canal. Through these mechanisms, stimulation of the vagus nerve, coupled with tonal stimuli, should enable the treatment of tinnitus. Experimental studies could prove the safety of the procedure, both for direct and transcutaneous stimulation [268–272].

While the first studies implanted a stimulation electrode on the neck, in a less invasive procedure an electrode was positioned transcutaneously on the auricle and stimulation of the vagus nerve was attempted for 30 min with a pulse rate of 25 Hz and an amplitude of 1–10 mA. During this time, the patients listened to music filtered at the tinnitus frequency. A total of 30 patients were treated; after 10 sessions, 50% reported relief from tinnitus. Side effects did not occur [273].

In 2014, an electrode was implanted cervically on the vagus for a study of tinnitus patients in the United States. The patients listened to music daily and were stimulated regularly. In 2017, this study was completed and 16 patients were treated in this way for 1 h daily for 1 year. They were compared with 14 patients who had also received implants, but for whom the therapy had only begun 6 weeks later. In these first weeks, the improvement of the tinnitus distress of the therapy group was 10% higher than in the control group; overall, the tinnitus distress of the treated group improved by 50%, that of the other group by 28%. The interpretation of the results classifies the improvement as significant, measured with the THI and other questionnaires. Tinnitus loudness was measured audiometrically. In addition, the Beck Depression Inventory (BDI) and an anxiety questionnaire were used. There were no significant changes according to the tables and graphs in any of the measured values. In the follow-up, subgroups were examined and it was found that the patients who did not have high-frequency tinnitus and noise-induced tinnitus seemed to benefit more. Finally, the authors state that vagus nerve stimulation paired with tones may be effective for subgroups of tinnitus patients, but this would need to be clarified in larger studies [274].

It has also been suggested that vagus stimulation could be supported by simultaneous administration of a muscarinic receptor type 1 to improve learning effects [275]. It has also been investigated whether cervical implanted vagus stimulation paired with tones alters speech understanding and influences voice and hearing functions. However, the study of seven tinnitus patients did not find any side effects of vagus stimulation in this regard [276].

A review from 2020 summarises all areas of application of invasive and transcutaneous vagus stimulation. The main areas of application are depression and epilepsy. Only a few studies can be claimed for tinnitus; results are not recorded and referenced [277].

4.1.8.3 Bimodal acoustic and electrical stimulation

##### Evidence-based recommendation

Bimodal acoustic and electrical stimulation should not be practiced for chronic tinnitus.

Strength of evidence: 2b (moderate); level of recommendation: recommendation (downgraded because of existing conflicts of interest)

Classification of consensus strength: strong consensus (100%), 1 abstention (conflict of interest)

Bimodal acoustic and electrical stimulation is safe to use, but robust evidence for efficacy is not available. A downgrading of the recommendation level of bimodal acoustic and electrical stimulation to a negative recommendation is made due to the lack of robust evidence of the studies and conflicts of interest of the authors in the publications listed in the evidence table.

The bimodal acoustic and electrical stimulation is supposed to simultaneously stimulate the auditory pathway and the trigeminal pathways thereby inducing plastic changes in the brain. This is based on animal experiments, for example, as described by Markovitz et al. [278].

In this therapy method, different acoustic stimuli adapted to the patient’s hearing ability are simultaneously given via headphones, and trigger points on the cheek and neck are electrically stimulated. This is intended to activate touch-sensitive nerves in the area of the auditory pathway. Since these parts of the auditory pathway are decisive for the amplification of tinnitus, which was established in animal experiments, success is also assumed in humans. In the pilot study after an animal study, 20 patients were then treated, in whom the tinnitus loudness and annoyance were reduced after 28 days. However, the effect only lasted 30 days. Treatment success is seen in a post hoc analysis, especially for patients with somatotinnitus [279, 280].

Another method combines the presentation of tones with electrostimulation of the tongue [281]. Again, only a pilot study or study protocol has been published to date, but this does not include a placebo group [282]. Although these seem to document general tolerability, valid and above all placebo-controlled studies are not yet available, but have been announced. The new study will include 192 patients who will be treated in four arms and followed up for 1 year. This study will also include an acoustic-stimulation-only arm without tongue stimulation [283]. In 2020, Conlon et al. [284] published the results of the study presented in 2017, unfortunately without placebo control. For this, 326 patients were recruited in two centres and randomly assigned to three equally sized stimulation groups each in a double-blind fashion. These groups differed in stimulus parameters and the sounds paired with them, whereby the first group used high-frequency sounds with synchronously applied electrical stimulation of the tongue, the second group used comparable stimuli without synchronisation, and the third group used acoustic stimulation with low-frequency sounds that were not synchronised with electrical tongue stimulation. Treatment was given for 12 weeks with 2 × 30 min of application daily. After treatment, the scores of all three groups improved significantly, the findings were measured with THI and TFI and checked again after 12 months, where they remained stable. The data of 20% of the participants did not improve with the bimodal stimulation. The evaluation does not differentiate which different hearing losses were present and especially why the values of all groups improved, although the stimulation parameters of the third group were relatively unsuitable for the postulate of neuroplastic changes and thus at least suggest a placebo effect. Side effects were described but were not severe. Furthermore, there is a considerable bias in the study (industry sponsorship bias, 11 of 13 authors had a conflict of interest in this respect)

4.1.8.4 Invasive electrical stimulation

##### Evidence-based recommendation

Invasive electrostimulation of the brain is to be omitted for chronic tinnitus.

Strength of evidence: 2a (no proof of efficacy); level of recommendation: strong recommendation (side effects)

Classification of consensus strength: strong consensus (100%)

For invasive tinnitus therapies, there is no evidence for their safe implementation or for therapeutic effects on tinnitus. Controlled studies and meta-analyses are lacking. Side effects can be severe. An upgrade of the recommendation level of invasive electrical stimulation to a strong negative recommendation is made because of the side effects.

Invasive forms of tinnitus treatment are so far solely experimental and include vagus nerve stimulation with an implantable electrode, constant electrical stimulation of the nervus vestibulocochlearis, extradurally implanted electrodes for brain stimulation and neural stimulators for deep brain stimulation. These are invasive methods that are not suitable for widespread and general use. Research in this area is limited to a few cases, and the pathophysiological mechanisms on which such therapies are based are neither sufficiently explored nor understood [285–288]. Controlled studies and meta-analyses are lacking; in addition, considerable side effects have been described in some of the few cases.

A review paper shows possible therapeutic perspectives through deep brain stimulation (DBS), in which an electrical pulse generator (brain pacemaker) is implanted in brain structures. The therapy has proven itself in the symptomatic treatment of otherwise therapy-resistant Parkinson’s disease and should now be examined concerning its possible use in tinnitus. This is based on considerations that in tinnitus patients, stimulation zones have been found in very different areas of the brain, which would then have to be controlled individually [289].

Anatomically and surgically targeted implantation of electrodes seems feasible if it would be therapeutically useful for tinnitus [290].

In a double-blind, placebo-controlled and randomised cross-over study, an electrode was implanted in the auditory cortex under general anaesthesia in nine patients with severely distressing unilateral tinnitus. This electrode was connected to a stimulator inserted in the pectoralis region. Patients were biphasically stimulated for 4 months, then randomised into two groups with either placebo or true stimulation. After a wash-out phase, cross-over stimulation was performed again. Therapy success was measured with a questionnaire (structured tinnitus interview—STI). In one patient the stimulator had to be explanted due to severe psychological decompensation and in three patients it was explanted after the end of the study, while five patients were still stimulated for another 3 years. During the open phase, the condition of five patients improved and that of two patients worsened. In the subsequent controlled phase, improvements were also seen, but in both the placebo and the directly stimulated group. No side effects were seen in any of the patients, in particular no hearing changes. The authors conclude from this study that direct electrostimulation of the auditory cortex is associated with a significant placebo effect, simply because of the surgery. However, there is no therapeutic effect on tinnitus distress when placebo effects are taken into account [291].

Six patients were stimulated with deep brain stimulation (DBS) at different positions of the caudate nucleus and were examined concerning a change in tinnitus. The control was carried out utilising functional magnetic resonance imaging, and they were compared with 14 patients matched according to TFI. A reduction in tinnitus loudness was achieved by stimulating five positions of the caudate nucleus, but not at 15 other positions. However, the changes only lasted for the duration of the stimulation [292].

In a similar study, six patients with therapy-resistant tinnitus had an electrode implanted in both caudate nuclei and were continuously stimulated for 24 weeks. One patient had to be explanted because of suicide risk; in the others, THI and TFI scores were evaluated. In three patients, the TFI improved by > 13 points, in four the THI by > 20 points; they were classified as responders. Side effects occurred but were reversible: postoperative pain, tinnitus worsening in all six participants, headache in five and milder side effects in two to three participants. There was no follow-up [293].

A review examined 21 studies since 2005 that invasively treated patients with severe and refractory tinnitus with various forms of neuromodulation. The studies were all of low quality due to small numbers of cases, lack of control groups and imprecise definition of outcome parameters. Although isolated successes have been reported, there is no evidence to support the use of invasive stimulation techniques as an alternative treatment option for chronic tinnitus [294].

4.1.8.5 Intracochlear electrical stimulation

See Cochlear implant (Sect. 4.1.2.3).

4.1.8.6 Transcutaneous electrical nerve stimulation

##### Evidence-based recommendation

Transcutaneous electrical nerve stimulation (TENS) should not be practiced for chronic tinnitus.

Strength of evidence: 2a (no proof of efficacy); level of recommendation: recommendation

Classification of consensus strength: strong consensus (100%)

For TENS, there is no or only moderate evidence for its safe implementation and therapeutic effects on tinnitus. Controlled studies and long-term observations are lacking.

In TENS treatment, interference currents with a frequency of 100–300 Hz are usually applied directly to the auricle or on the mastoid. The electrodes are applied like a band-aid or held in place during treatment. The treatment devices, usually used for orthopaedic complaints, can be purchased by the patient. Scientific studies have been published only sporadically, so far without scientific evidence.

In 2014, Lee et al. [295] studied 65 patients with chronic tinnitus, 45 of whom received TENS treatment and 20 of whom only received sham treatment. Therapy success was measured with the THI and VAS. Subjective improvement was experienced by 62.2%, especially those patients who had low-frequency tinnitus and mild hearing loss, but relief lasted only 1 month.

In 2019, Li et al. [296] treated 46 patients with acute tinnitus: 23 with TENS, and 23 with sham treatment. Therapy success was measured with the TQ and THI; there was a just significant improvement of *p* < 0.01 after 4 weeks compared to placebo treatment.

Tutar et al. [297] treated 60 patients with chronic tinnitus in ten TENS sessions every 4 days. The patients were divided into three groups: 20 patients each were treated on one ear, both ears, or had only sham treatment. The treatment was carried out with a frequency of 200 Hz and 10–30 mA. The values of both treatment groups improved significantly (*p* < 0.05) compared to the non-treatment. But the values of the control group also improved, albeit less. The authors therefore also emphasise a placebo effect.

Feasibility studies, reliable therapy control studies or meta-analyses with larger numbers of cases are not available for TENS treatment.

A meta-analysis of this treatment assessed 17 studies with 1215 participants, but only four studies could be evaluated for a meta-analysis. There were significant improvements in THI scores and VAS values. The complaints of 40% of the patients improved for at least 3 months [298].

4.1.8.7 Low-level laser therapy

##### Evidence-based recommendation

Low-level laser therapy should not be practiced for chronic tinnitus.

Strength of evidence: 2b (no evidence of efficacy); level of recommendation: recommendation

Classification of consensus strength: strong consensus (100%)

For low-level laser therapies, there is no evidence for their safe implementation or for therapeutic effects on tinnitus. Controlled studies and meta-analyses are lacking.

In so-called low-level laser therapy (LLLT—formerly: ‘soft laser’), which has been marketed since 1985, the ear, mastoid or auditory canal is irradiated with low intensity (approx. 100 mW) and a wavelength of usually 830 nm for 15–20 min over several days. Work on anatomical specimens in the past has shown that this irradiation cannot reach the inner ear or even higher structures of the auditory pathway. There has been no scientific evidence, but occasionally new studies have been presented that use this form of therapy.

In 2015, Dehkordi et al. [299] concluded that after treatment of 66 tinnitus patients, of whom 33 received real and 33 received sham irradiation, no differences were found between the groups; in both groups the suffering from tinnitus improved slightly (*p* < 0.589 in TSI).

Choi et al. [300] also saw no significant improvement in 2019 after treatment of 38 patients (19 real, 19 sham): no significant improvements and no placebo effects.

#### 4.1.9 Manual medical and physiotherapeutic therapy

##### Evidence-based recommendation

Manual medical and physiotherapeutic therapy should be offered for chronic tinnitus if modulations of the tinnitus are present due to comorbid changes in the cervical spine and masticatory system.

Strength of evidence: 1b (high); level of recommendation: recommendation

Classification of consensus strength: consensus (79%)

Manual medical and physiotherapeutic therapies should be used if tinnitus modulations have been determined in the course of the orienting basic examination of the cervical spine and the masticatory apparatus, and findings have been determined through further manual medical examination, which argue for an involvement of the cervical spine, temporomandibular joint function and muscular trigger points/dysbalances.

Manual–medical and physiotherapeutic therapies have a positive effect on the degree of tinnitus severity and complaints in the area of the cervical spine. A combination of physiotherapeutic and manual therapy in addition to patient’s education has positive effects on tinnitus patients with concomitant craniomandibular dysfunction.

A gradation of the level of recommendation is made in the evidence table due to the low methodological rigour and heterogeneity of the RCTs.

A recent systematic review [77] lists three RCTs and records positive results for the application of physiotherapeutic/manual techniques in the cervical spine, including cervical mobilization, myofascial techniques and osteopathy. However, the methodological quality of the included studies is low. Another systematic review [301] includes two RCTs on cervical spine treatments. Cervical spine therapy can record positive effects concerning the severity of tinnitus and includes physiotherapeutic exercises and trigger point treatment. The methodological quality of the RCTs included is again low. In addition, a current RCT [302] with 61 participants concludes that a combination of physiotherapeutic and manual therapy (exercises for craniocervical and temporomandibular joints as well as myofascial techniques) together with patient education offers a significantly better outcome concerning tinnitus severity than physiotherapy with education alone.

#### 4.1.10 Nutritional supplements

##### Evidence-based recommendation

Food supplements are to be omitted for chronic tinnitus.

Evidence strength: 1c (no proof of efficacy); level of recommendation: strong recommendation

Consensus strength classification: strong consensus (100%)

Based on RCTs, there is no evidence that dietary supplements (e.g. vitamins, minerals or phytotherapeutics) have a proven efficacy on tinnitus. There is no evidence that it differs from placebo treatment in terms of tinnitus reduction or side effects.

Several nutritional supplements have been used for tinnitus, including lipoflavonoids, garlic, homeopathy, traditional Chinese–Korean herbal medicine, honeybee larvae, and various vitamins and minerals. Evidence of the efficacy of these therapies for tinnitus is not available [232]. A more recent review also finds that there is no evidence of dietary supplements on tinnitus. In particular, a reduction of salt or caffeine had neither a positive nor a negative effect on tinnitus perception [303]. In a review by Wegner et al. [226], five studies with approx. 300 tinnitus patients each were examined concerning betahistine administration versus placebo (vitamin B_12_), and no effect (−0.16) on tinnitus loudness and impairment was found after 12 weeks.

#### 4.1.11 Acupuncture

##### Evidence-based recommendation

(Electro)acupuncture should not be practiced for chronic tinnitus.

Strength of evidence: 1c (no proof of efficacy); level of recommendation: recommendation

Classification of consensus strength: strong consensus (100%)

Based on RCTs, there is no evidence that acupuncture or electroacupuncture have proven efficacy on tinnitus. There is moderate evidence that they can improve comorbidities such as tension or pain with a possible positive effect on tinnitus.

Concerning the effect of acupuncture in patients with chronic tinnitus, no recommendation can be made due to the existence of qualitatively insufficient studies; a benefit cannot be demonstrated, and minimal harm is assumed.

A systematic review of acupuncture for the treatment of tinnitus included nine RCTs with a total of 431 participants [304]. This systematic review highlighted the heterogeneity of study designs as well as their methodological limitations using the Cochrane bias risk assessment tool. There were variations in study design of the types of acupuncture interventions, frequency, intensity and duration of treatments, selection of other control groups, as well as variability with blinding and selection of outcome measures, many of which were not validated [303]. The authors concluded that the small number of RCTs of acupuncture for the treatment of tinnitus with small sample sizes and methodological issues is insufficient to conclude effectiveness.

Recent reviews (18 studies with 580 tinnitus patients [305] and five studies with 182 tinnitus patients [306]) show a weak effect of (electro)acupuncture compared to the various comparison groups. However, the methodological limitations described above still apply, and thus no reliable conclusions on the effectiveness of acupuncture are possible.

#### 4.1.12 Self-help

##### Evidence-based recommendation

Patients with chronic tinnitus should be motivated to participate in self-help programmes.

Strength of evidence: 2b (moderate); level of recommendation: recommendation

Classification of consensus strength: strong consensus (100%)

Self-help is an effective and supportive aspect of treatment for many people affected.

**Preamble to self-help**

Despite some studies on the significance and functionality of self-help (SH) for tinnitus, there is little evidence or no knowledge of whether and how SH has a specific effect on tinnitus. Presumably, this is because the largest part of SH is carried out voluntarily or autonomously out of one’s pocket in the form of membership fees and indirect costs such as the time spent personally.

Since nothing can be sold or earned here, no lobby would encourage studies on the evidence of SH.

What is SH? If we want to be consistent with the term, in the true sense of the word, SH is when those affected help each other: Mostly this happens in face-to-face SH groups (SHG) whose meetings are more or less structured with a fixed core and changing participants or who contact each other in internet forums.

However, the term SH moves away from the idea of SH when working through the tinnitus issue oneself with the help of a ‘self-help book’ or ‘internet-based self-help’ or increasingly with tinnitus apps misleadingly counted as the original SH.

If minimal-contact interventions with experts are integrated into the latter forms of intervention, it is a matter of treatment/therapy and no longer of SH.

**Why recommend SH interventions?**

In healthcare reforms, the stronger involvement of patients in their healthcare is a common theme. Self-help is commonly understood as seeing tinnitus sufferers as active partners in their healthcare, taking responsibility for their well-being. It is an integral part of the UK Department of Health tinnitus management guidelines [225], the US tinnitus practice guideline [307], the American Academy of Otolaryngology (AAO; http://otojournal.org) and the American Tinnitus Association ([308]; ATA; McGinnis 2001; www.ata.org).

Despite many publications on the importance and functionality of SHG activity, there is little evidence on whether and how SH specifically affects tinnitus [308, 309].

Only recently, a study on the effect of SH was published [310]. At the time of the survey, 800 of the 13,000 members of the non-profit SH organisation Deutsche Tinnitus-Liga (DTL) were active in SH groups. In a cross-sectional study, the tinnitus distress (Mini-TQ; [64]) was assessed in 1108 affected persons (mean 61 years, 60% men) using valid items of the Structured Tinnitus Interview (STI; [310]) on tinnitus knowledge (TK), tinnitus coping and quality of life (QoL; WHOQOL-BREF 1998). As a result, the regression analyses show highly significant correlations of community SH activity with TK, coping as well as health system orientation and self-confidence, but no significant differences concerning general QoL.

Recently, SH activity was shown as a gradient in the four groups of current SHG participation (*n* = 217), previous SHG participation (*n* = 118), DTL membership without SHG participation (*n* = 641) and no references to SHG or DTL (*n* = 132). The former and latter groups thus show the greatest differences and most clearly demonstrate the benefits of SHG participation [310]. When controlled logistic regressions are determined for age, gender, education, equivalised income and tinnitus distress, odds ratios of 6.94 for TW, 3.83 for knowledge of help options, and 7.75 for self-confidence in knowing more about tinnitus than most doctors are found between these two groups. The other two groups are in between with corresponding and also significant gradations. Despite all the methodological limitations of this cross-sectional study, it seems more likely that tinnitus-related knowledge and other benefits are a result of SHG participation than vice versa.

**Smartphone apps as guided SH?** A plethora of apps for tinnitus can be found, which in the broadest sense of the development of digital coping forms can be attributed to Internet programmes and thus also categorised as guided SH [312]. The advantage of the apps is that they are installed on the smartphone and, like SH books, can be conveniently used anytime and anywhere, partly offline and partly online. In a review, Mehdi et al. [313] identified and evaluated 34 systematically recorded apps for tinnitus. Although all of them were considered to have a certain technical functional value, they all lacked scientific evidence. Nagaraj and Prabhu [167] came to similar conclusions. In the course of the current expansion of the Digitalisation Act of the Federal Republic of Germany, the Federal Institute for Drugs and Medical Devices (BfArM) is attempting, according to § 139e of the German Social Law Book V (SGB V), to bring the exploding variety of health apps into regulated channels by the authorities (Digital Health Applications, DiGA, https://www.bfarm.de/DE/Medizinprodukte/DVG/_node.html).

In the field of health services, the spread of digitalisation (telemedicine; [314]) has resulted in increasingly inconsistent terminology, the keywords of which provoke considerable miscommunication for SH. Thus, under laboratory conditions of research, partly inconsistent word combinations such as ‘self-help via internet’, ‘internet-based self-help treatment’, ‘internet-guided self-help’, ‘internet-based therapeutic self-help intervention’ and ‘internet-based self-help training’ etc. have been developed and evaluated for various psychotherapy procedures, which, in contradiction to their taxonomy, lack any reference to SH [315, 316]. A correct taxonomy, e.g. CBT, would be ‘Healthcare interventions delivered over the internet’ [317] or ‘internet-delivered CBT’ [318].

However, if there is no consensus on terminology among researchers, it is very difficult for guideline writers, and not possible at all for media, to report correctly on SH versus therapy and to convey an accurate message to the public. A common and clearly defined terminology would undoubtedly help to navigate this [316].

**Implications for practice**: Service providers should inform their patients about SH options analogous to the recommendations mentioned at the beginning and encourage them very early on to consider an SHG as a possible option for their self-management [307].

To avoid blatant misunderstandings, internet-based therapy programmes including apps with or without expert support should under no circumstances be terminologically declared as SH.

### 4.2 Conclusion

**Fig. 1:**
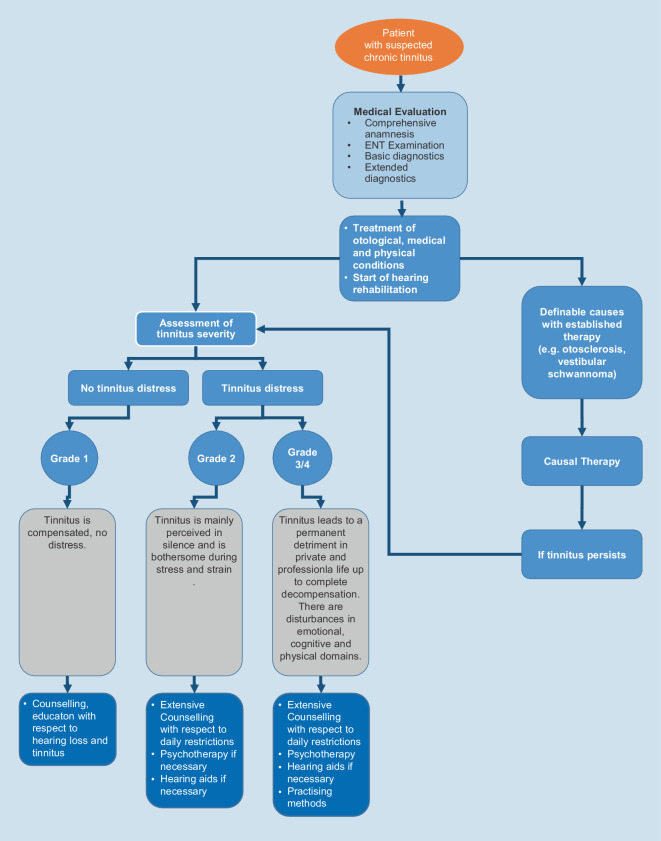
Treatment algorithm for chronic tinnitus

The treatment of chronic tinnitus is based on well-founded diagnostics with the assessment of both the audiological characteristics of the ringing in the ears and any existing hearing loss as well as the psychosomatic comorbidities and other concomitant diseases. This should be the basis for detailed and reassuring counselling. In addition to counselling, tinnitus-specific cognitive-behavioural therapy and well-founded psychotherapeutic interventions are available for further treatment as an individual or group design, individually or multimodally. They improve tinnitus distress, quality of life and can also have an impact on comorbidities such as anxiety and depression (see Fig. 1).

In preparation for tinnitus-specific psychotherapy, it is important to guide patients and not leave them alone with the ear noise due to the self-perceived therapeutic powerlessness (helplessness, loss of control). Rather, the patient is told that gradual habituation to the ringing in the ears can often be achieved through psychotherapy. The patient is informed that the doctor will carry out this therapy or that the patient will be referred to a tinnitus specialist, to a psychosomatic or a psychotherapeutic doctor who is qualified in tinnitus-specific psychotherapy or a psychological psychotherapist in appropriately qualified practices (medical practice, psychological psychotherapy) or facilities (e.g. specialised clinic or centre with guideline-oriented therapy implementation). In this context, good teamwork among the therapists involved is desirable, ideally within the framework of a tinnitus conference.

The goal of meaningful tinnitus therapy is that the tinnitus should in all likelihood no longer play a significant role in the daily life routine. An additional therapeutic task is to make patients aware of the need to be prepared to participate in the therapy themselves and extensively in the context of psychotherapy.

A tinnitus symptom-related drug therapy is not available.

The following measures are useful:

1. Counselling
2. Tinnitus-specific psychotherapy (individual or multimodal)
3. Compensation of hearing loss (hearing aids)
4. Co-treatment of comorbidities (see Table 1), then possibly also psychopharmaceuticals

Hearing aids should be recommended for the treatment of concomitant hearing loss. In cases of severe hearing loss or deafness, even unilateral, a cochlear implant should be indicated, for there is also good evidence concerning tinnitus habituation.

Tinnitus-specific psychotherapies can be carried out in appropriately qualified facilities such as practices, tinnitus centres, clinics or rehabilitation facilities. Individual or group therapies can be carried out or these can be combined as a multimodal approach. The individually pronounced comorbidities can be targeted at the same time. Internet-supported psychotherapeutic interventions, with and without direct therapist contact, have already been evaluated in isolated cases or are still being further researched.

Polypragmatic tinnitus treatments should be rejected if therapy methods are used whose effectiveness has not been proven in controlled studies.

Minority opinion of the DGPPN on the last paragraph of the conclusion:

If a patient has psychological comorbidities (e.g. depression, anxiety), then a guideline-compliant treatment of these comorbidities should take place within the framework of the overall treatment of tinnitus by appropriately qualified disciplines with the involvement of psychiatry and psychotherapy or psychosomatics. If outpatient treatment is not possible due to the severity of the distress, partial inpatient or inpatient therapy can take place in appropriately qualified facilities.

Justification: If patients with tinnitus have a psychiatric illness, then the relevant specialist disciplines should be involved in the treatment.

If inpatient treatment is necessary due to psychiatric comorbidity, the relevant disciplines (psychiatry and psychotherapy, psychosomatics) should also be involved in the inpatient treatment.

If a patient with severe decompensation due to tinnitus-induced helplessness or severe comorbidities (depression, anxiety) cannot be treated as an outpatient, partial inpatient or inpatient therapy can be recommended if it is individualised, interdisciplinary and multimodal for patients with significant psychosomatic comorbidity.

However, the recommendation only applies if the therapy includes the evidence-based treatment procedures specified above. Inpatient therapy aims to compensate for the helplessness with the means of a hospital until a reduction in the level of suffering is achieved. However, the typical procedures of the hospital may be necessary as long as compensation of the helplessness of the patient is required.

According to the available studies, there is no evidence for special devices developed and advertised for the treatment of tinnitus as well as acoustic stimulation with tones, sounds or alienated music. This also applies to invasive procedures such as brain stimulation.

## 5. Appendix

### 5.1 Appendix 1

Figure 1.

### 5.2 Appendix 2: Counselling for Tinnitus

The doctor will not determine the course of the conversation by his or her questions but will allow the patient to describe his or her complaints and ideas about the disease spontaneously and in detail. The doctor learns that the patient’s hypotheses about the illness are mostly inaccurate, but are sometimes perceived by the patient as extremely threatening. The threatening nature of the (false) tinnitus hypothesis accounts for a significant part of the illness value of tinnitus for many patients. Some of the patients do not believe in the existence of tinnitus until they receive tinnitus counselling, while at the same time they subjectively feel threatened by it. These patients also feel left alone and abandoned.

In particular, life situations will be addressed in which the tinnitus is perceived as disturbing (work, leisure, rest, falling asleep, tense situations), but also as bearable (background noise, music, the sound of the sea, fountains, machine noise, general distraction, use of hearing aids, drinking alcohol, etc.).

The medical interview will be based on the patient’s description and will include the following explanations:

- There are phantom sounds that others, including the doctor, do not perceive.
- There are physiological noises such as swallowing, which are usually louder than the subjective ear noise, but which are not perceived.
- The patient suffers from such noises and the doctor believes this.
- Help for the sick person is almost always possible; a cessation of the chronic ringing in the ears is possible even after years (up to 27%). In the chronic stage, the goal of ‘eliminating the tinnitus’ is counterproductive. The primary goal should be habituation, which secondarily enables the path to complete ‘forgetting’ of the tinnitus.
- In the case of worsening, a wide range of treatment options are available.
- Education to understand the correct disease model: Based on the examination results, the doctor can tell the patient that the noises are not an expression of a brain tumour or similar, there is no danger to life, no danger of apoplexy or danger of any other brain disorder. Rather, the noises come from the ear or the hearing system. In the session, the patient is introduced to the basic anatomy and physiology of the hearing system, if possible with the help of illustrations. Based on this, the patient is presented with her/his individual tinnitus disease model, which includes her/his history, findings and aspects of the development and maintenance of symptoms. Patients can then reduce anxiety regarding tinnitus when they understand for themselves that they are not suffering from a dangerous disease of the ear and brain.
- Advice on sound enrichment: The focus is on avoiding silence. Several ways are available. Enrich sound in the daily environment pleasantly. Irritating or disturbing sounds must be avoided at all costs. The best sound signals are sound events in nature. In summer, one may simply open the window if the sound environment of the house is perceived as pleasant by the patient. Most people find the sounds of nature relaxing. Tinnitus (and sometimes hyperacusis) patients find forests, gardens or beaches pleasant places to be and also like to hear rain and wind. For other patients, the pleasant sound of a fan or table fountain may be suitable in summer. Often, sustained sound enrichment means using CDs that produce white noise, physiological noise or volume-modulated noise (wave noise) for several hours at a time (not a volume that leads to tinnitus suppression; the sound signal must be level and pleasantly audible).
- If necessary, advice on hearing aids, assessment of communication impairment and separation of symptom areas: Complaints about communication impairments are almost always due to a co-existing hearing loss and not to tinnitus. Early acceptance of hearing aid fitting can quickly and significantly reduce tinnitus sensitisation. One of the mechanisms is probably the increased attention to speech signals while simultaneously turning away from the tinnitus.

### 5.3 Appendix 3: Medical history questions and clinical severity classification

The following questions are relevant (based on the STI, [318]):

- In which ear do you hear your tinnitus (right, left, both sides, head)?
- When did your tinnitus start (right, left)?
- Did your tinnitus start suddenly or slowly (right, left)?
- What cause(s) do you suspect for the development of the tinnitus?
- Is your tinnitus only audible in silence?
- Can the tinnitus be masked by ordinary ambient noise?
- Does your tinnitus drown out all sounds?
- Is the volume of your tinnitus always the same or does it fluctuate throughout the day?
- Does normal environmental noise make your tinnitus louder?
- Is your tinnitus constant during the day? Are there interruptions, and if so, for how long?
- Is your tinnitus distressing?
- Is your tinnitus distressing? From the beginning or only later?
- Are you particularly sensitive to noise?
- Can you influence the tinnitus through self-directed measures such as shifting attention, relaxation or other means?
- Do you or other people notice that you hear or understand worse?
- Has the tinnitus occurred together with hearing loss and/or ear pressure?
- Do you have balance problems?
- Has the tinnitus occurred together with severe spinning vertigo?
- Can the tinnitus be influenced by certain head postures or jaw movements?
- Can the tinnitus be influenced by certain jaw/chewing muscle tension?
- Can the tinnitus be influenced by physical exertion?
- What medication are you currently taking?
- Have you been treated with medication for serious infections (e.g. tuberculosis, meningitis, myocarditis, pneumonia, malaria, etc.) or malignant diseases and if so, with what?
- Have you been irradiated because of a malignant disease in the head and neck area?
- Do you have any cardiovascular or metabolic diseases?
- Are there any indications of other disorders and comorbidities (see Table 1)?

**Severity classification of tinnitus**

The determination of the severity of tinnitus is important and recommended for the therapy indication in individual cases. The classification of the degree of severity according to Biesinger et al. [65] is a pragmatic classification oriented to the clinical situation and takes into account the effect of the ear noise in the professional and private sphere:

- Grade 1: The tinnitus is well compensated, no suffering.
- Grade 2: The tinnitus mainly appears in silence and is disturbing in the case of stress and strain.
- Grade 3: Tinnitus leads to permanent impairment in the private and professional sphere. Disturbances occur in the emotional, cognitive and physical spheres.
- Grade 4: The tinnitus leads to complete decompensation in the private sphere and occupational disability.

The evidence tables for the treatment procedures examined in this guideline and the methodological details can be found on the AWMF website [321].

## Abbreviations

A: Artery
ACI: Attention Control Index
ACT: Acceptance and Commitment Therapy
ATQ: Automatic Thoughts Questionnaire
BDI: Beck Depression Inventory
CBT: Cognitive Behavioural Therapy
CBT‑T: Cognitive Behavioural Therapy plus Tinnitus education
CCEI: Crown–Crisp Experiential Index
CET: Coping Effectiveness Training
CFQ: Cognitive Failures Questionnaires
CR: Cognitive Restructuring
DBS: Deep Brain Stimulation
HADS: Hospital Anxiety and Depression Scale
HADS‑A: Hospital Anxiety and Depression Scale—Anxiety subscale
HADS‑D: Hospital Anxiety and Depression Scale—Depression subscale
HHIA‑S: Hearing Handicap Inventory for Adults Screening version
HQ: Hyperacusis Questionnaire
HRD/HRSD: Hamilton Rating Scale for Depression
HUI: Health Utilities Index
iCBT: Internet-based CBT
ISI: Insomnia Severity Index
IWDA: Interference with Daily Activities
M: Morbus
MBCT: Mindfulness-Based Cognitive Therapy
MSPQ: Modified Somatic Perception Questionnaire
N: Nervus
N: Nucleus
NHS: National Health Service (UK)
PHQ‑9: Patient Health Questionnaire‑9
QIPA: Tinnitus Psychological Impact Questionnaire
QoLI: Quality of Life Inventory
RCT: Randomised Controlled Trial
RT: Relaxation Therapy
SD: Standard Deviation
STAI: State Trait Anxiety Inventory
SWLS: Satisfaction With Life Scale
TAQ: Tinnitus Acceptance Questionnaire
TCQ: Tinnitus Cognitions Questionnaire
TCS: Tinnitus Catastrophizing Scale
TCSQ: Tinnitus Coping Strategies Questionnaire
tDCS: Transcranial Direct Current Stimulation (*Transkranielle Gleichstromstimulation*)
TENS: Transcutaneous Electrical Nerve Stimulation
TEQ: Tinnitus Effects Questionnaire
TFI: Tinnitus Functional Index
THI: Tinnitus Handicap Inventory
THI‑S: Tinnitus Handicap Inventory Screening version
THQ: Tinnitus Handicap Questionnaire
TK: Tinnitus Knowledge
TKQ: Tinnitus Knowledge Questionnaire
TQ: Tinnitus Questionnaire
TRQ: Tinnitus Reaction Questionnaire
TRT: Tinnitus Retraining Therapy
VET: Veterans
WHO QoL: World Health Organization Quality of Life

## Declarations

For this article no studies with human participants or animals were performed by any of the authors. All studies mentioned were in accordance with the ethical standards indicated in each case.
